# NADH inhibition of SIRT1 links energy state to transcription during time-restricted feeding

**DOI:** 10.1038/s42255-021-00498-1

**Published:** 2021-12-13

**Authors:** Daniel C. Levine, Hsin-Yu Kuo, Hee-Kyung Hong, Jonathan Cedernaes, Chelsea Hepler, Alexandra G. Wright, Meredith A. Sommars, Yumiko Kobayashi, Biliana Marcheva, Peng Gao, Olga R. Ilkayeva, Chiaki Omura, Kathryn M. Ramsey, Christopher B. Newgard, Grant D. Barish, Clara Bien Peek, Navdeep S. Chandel, Milan Mrksich, Joseph Bass

**Affiliations:** 1 Department of Medicine, Division of Endocrinology, Metabolism, and Molecular Medicine, Feinberg School of Medicine, Northwestern University, Chicago, IL USA; 2 Departments of Chemistry, Biomedical Engineering, and Cell and Molecular Biology, Northwestern University, Evanston, IL USA; 3 Departments of Medical Sciences and Medical Cell Biology, Uppsala University, Uppsala, Sweden; 4 Robert H. Lurie Cancer Center Metabolomics Core, Feinberg School of Medicine, Northwestern University, Chicago, IL USA; 5 Duke Molecular Physiology Institute, Department of Medicine, Division of Endocrinology, Metabolism and Nutrition, Duke University School of Medicine, Durham, NC USA; 6 Department of Biochemistry and Molecular Genetics, Feinberg School of Medicine, Northwestern University, Chicago, IL USA; 7 Department of Medicine, Feinberg School of Medicine, Northwestern University, Chicago, IL USA

**Keywords:** Acetylation, Metabolic syndrome, Metabolism

## Abstract

In mammals, circadian rhythms are entrained to the light cycle and drive daily oscillations in levels of NAD^+^, a cosubstrate of the class III histone deacetylase sirtuin 1 (SIRT1) that associates with clock transcription factors. Although NAD^+^ also participates in redox reactions, the extent to which NAD(H) couples nutrient state with circadian transcriptional cycles remains unknown. Here we show that nocturnal animals subjected to time-restricted feeding of a calorie-restricted diet (TRF-CR) only during night-time display reduced body temperature and elevated hepatic NADH during daytime. Genetic uncoupling of nutrient state from NADH redox state through transduction of the water-forming NADH oxidase from *Lactobacillus brevis* (*Lb*NOX) increases daytime body temperature and blood and liver acyl-carnitines. *Lb*NOX expression in TRF-CR mice induces oxidative gene networks controlled by brain and muscle Arnt-like protein 1 (BMAL1) and peroxisome proliferator-activated receptor alpha (PPARα) and suppresses amino acid catabolic pathways. Enzymatic analyses reveal that NADH inhibits SIRT1 in vitro, corresponding with reduced deacetylation of SIRT1 substrates during TRF-CR in vivo. Remarkably, *Sirt1* liver nullizygous animals subjected to TRF-CR display persistent hypothermia even when NADH is oxidized by *Lb*NOX. Our findings reveal that the hepatic NADH cycle links nutrient state to whole-body energetics through the rhythmic regulation of SIRT1.

## Abstract (web summary)

Using a model of time-restricted feeding in mice, Levine et al. show that the hepatic NADH cycle links nutrient state to whole-body energetics through the rhythmic regulation of SIRT1.

## Main

Metabolic homoeostasis in mammals is mediated by interlocked nutrient-sensing and temporal signals throughout the 24-h light/dark cycle. The molecular clock network drives oscillations of a broad range of transcripts and metabolites that direct anabolic and catabolic metabolism in anticipation of the fasting/feeding–sleep/wake cycle^1–3^. Interactions between the core molecular clock and nutrient-responsive transcription factors (TFs) contribute to metabolic homoeostasis, yet how these pathways cooperate under long-term energy-deficient conditions has remained obscure.

In yeast, calorie restriction (CR) transcriptionally reprogrammes metabolic gene expression to shift oxidative fuel preference and maintain energetic homoeostasis of the cell^4^. In mammals, CR downregulates energy-intensive processes, such as thermogenesis during sleep, and upregulates anabolic processes within the liver that convert metabolite stores into energetic substrates for the brain^5,6^. NAD^+^ and the NAD^+^-dependent ySIR2p deacetylase are required for the transcriptional response to CR in yeast^7^, although the role of NAD^+^ in the response to low-energy conditions in mammals remains less clear.

Transcriptomic studies have revealed robust oscillations in the expression of the rate-limiting enzyme involved in NAD^+^ biosynthesis across peripheral tissues, which in turn feedback to regulate metabolic transcription cycles through SIRT1-mediated deacetylation and inhibition of the circadian repressor PER2 (refs. ^8–13^). Supplementation of NAD^+^ with the soluble precursor nicotinamide riboside in ageing mice reverts senescence of the sleep/wake and mitochondrial oxidative respiration cycles, suggesting that robustness of the NAD^+^–SIRT1-clock pathway may enhance fitness^10^. NAD^+^ also functions as an electron shuttle in oxidoreductase equilibrium reactions that vary according to both nutrient availability and time of day^14,15^, yet whether metabolic control of rhythmic transcription is modulated by cyclic changes in NAD(H) balance remains unknown.

To investigate whether NAD(H) balance provides a signal to regulate transcriptional and metabolic cycles in vivo, we examined the effect of uncoupling nutrient state from NADH levels by tonically inducing NADH oxidation during CR through hepatic transduction of *Lb*NOX^16,17^. When NADH levels were dissociated from energy state using *Lb*NOX, we observed transcriptional reprogramming of SIRT1- and BMAL1-mediated gene networks that regulate fatty acid and amino acid metabolism and thermogenesis. Our studies reveal that NADH redox state drives energy conservation during sleep through rhythmic inhibition of SIRT1 and downstream circadian processes.

## Results

### Elevation in hepatic NADH drives morning torpor with TRF-CR

**Fig. 1:**
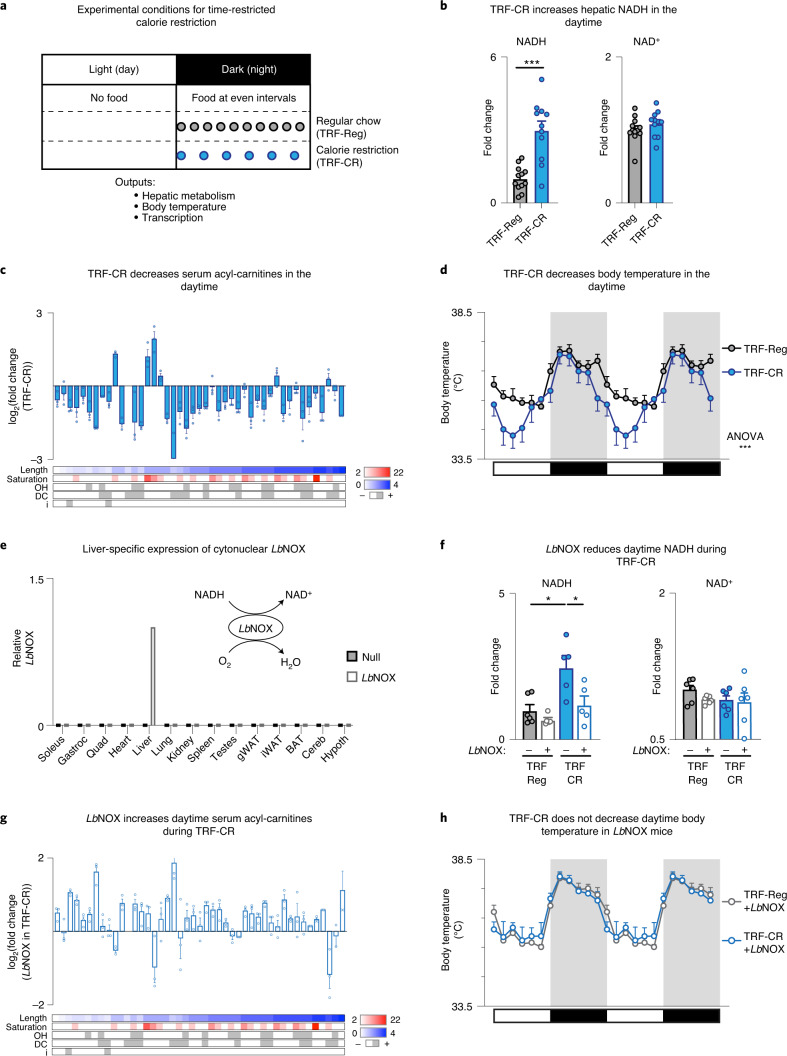
Elevated NADH drives the daytime dip in body temperature during time-restricted calorie restriction. **a**, Model for the TRF-CR diet using an automated feeder system in 4–6-month-old male C57BL/6J mice. Control mice received a 300 mg pellet of regular chow every 1.2 h throughout the dark period (TRF-Reg), whereas TRF-CR mice received a 300 mg pellet of a carbohydrate-depleted, nutrient-controlled chow every 2 h throughout the dark period, resulting in a 40% reduction in calories. **b**, Relative concentration of NADH and NAD^+^ by HPLC in liver of TRF-Reg (*n* = 12) and TRF-CR (*n* = 11) mice during the daytime (ZT4). **c**, log_2_(fold change) in daytime serum acyl-carnitine levels during TRF-CR compared with TRF-Reg mice (*n* = 3). **d**, Body temperature rhythms monitored non-invasively using subcutaneous probes in TRF-Reg (*n* = 12) and TRF-CR (*n* = 9) mice over 24 h (double plotted for clarity). **e**, Model depicting NADH-consuming reaction of *Lb*NOX. Representative tissue-specific expression profile of cytonuclear *Lb*NOX in null- and *Lb*NOX-transduced mice relative to *Lb*NOX-transduced liver. **f**, Relative concentration of NADH and NAD^+^ in liver of null- and *Lb*NOX-transduced TRF-Reg (*n* = 6) or TRF-CR (*n* = 5 for NADH, *n* = 6 for NAD^+^) mice during the daytime. **g**, log_2_(fold change) in daytime serum acyl-carnitine levels in TRF-CR mice transduced with cytonuclear *Lb*NOX compared with TRF-CR mice transduced with null virus (*n* = 3). **h**, Body temperature rhythms in *Lb*NOX-expressing TRF-Reg (*n* = 12) or TRF-CR (*n* = 9) mice over 24 h (double plotted for clarity). Data are presented as mean ± s.e.m. Statistics were performed﻿ with unpaired, two-tailed Student’s *t* test unless otherwise noted in the figure. *P < 0.05, ***P < 0.001. ANOVA, analysis of variance; Cereb, cerebellum; DC, dicarboxylate; Gastroc, gastrocnemius muscle; gWAT, gonadal white adipose tissue; Hypoth, hypothalamus; i, isomer; iWAT, inguinal white adipose tissue; OH, hydroxy; Quad, quadriceps muscle.

**Extended Data Fig. 1:**
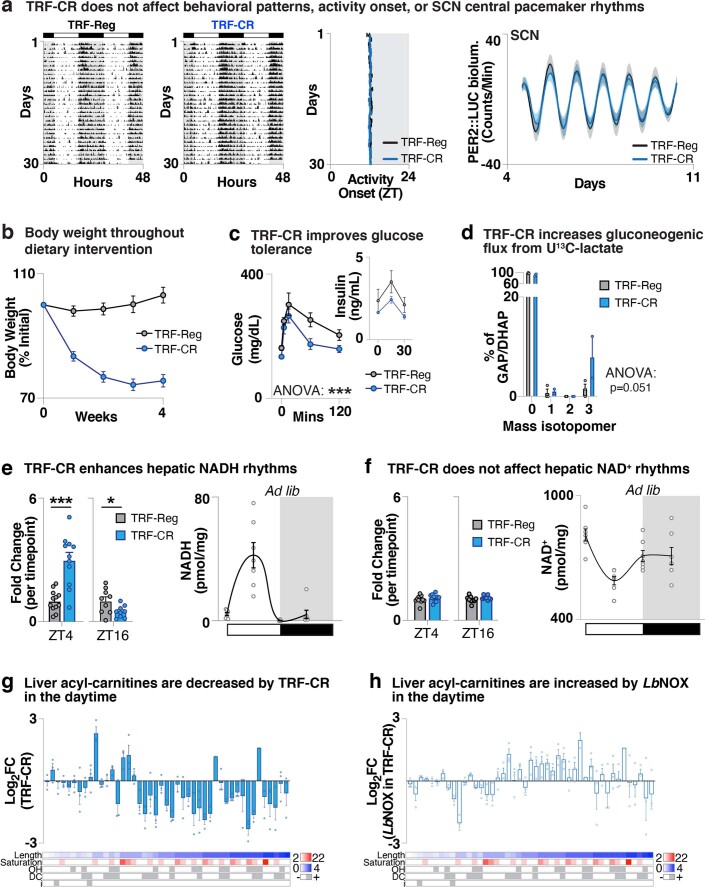
Circadian and metabolic profiling in TRF-CR mice. **a**, (*left*) Representative wheel-running behaviour and (*middle*) quantification of average daily onset of activity for TRF-Reg (n = 5) and TRF-CR (n = 5) mice throughout the duration of the 4-wk intervention (double plotted for clarity). (*right*) Background-corrected PER2::LUCIFERASE readings from excised suprachiasmatic nucleus (SCN) of TRF-Reg (n = 6) and TRF-CR (n = 6) mice. The grey shading indicates mean values ± SEM. Experiments were performed in 4–6 mo old male C57BL6/J mice. **b**, Body weight of TRF-Reg (n = 15) and TRF-CR (n = 15) mice over the duration of the 4-wk study. **c**, Blood glucose and serum insulin during oral glucose tolerance testing performed during the daytime (at ZT4) in TRF-Reg (n = 6) and TRF-CR (n = 6) mice. **d**, GAP/DHAP mass isotopomer distribution determined by mass spectrometry of liver from TRF-Reg (n = 3) and TRF-CR (n = 2) mice 30 min following i.p. administration of a 10:1 mix of U^13^C-lactate:U^13^C-pyruvate (1 g/kg). **e-f**, (*left*) Relative concentration of (e) NADH and (f) NAD^+^ by HPLC in liver of TRF-CR compared to TRF-Reg mice during the day (ZT4) (n = 12 for TRF-Reg, n = 11 for TRF-CR,) and at night (ZT16) (n = 9 for each diet) for each time point. (*right*) Concentration of (e) NADH and (f) NAD^+^ in *ad lib* fed wild-type mouse liver by HPLC every 6 hrs for 24 hrs (n = 7). **g-h**, Log_2_-FC in daytime liver acyl-carnitine levels in (g) TRF-CR compared to TRF-Reg mice and (h) *Lb*NOX- compared to null-overexpressing TRF-CR mice (n = 3). Data are presented as mean values ± SEM. Statistics were performed with unpaired, two-tailed student’s t test except as otherwise noted in the figure. *p < 0.5, ***p < 0.01.

We first sought to examine how changes in energy state during a TRF-CR diet impact NAD^+^ and NADH across the fasting/feeding cycle compared with time-restricted feeding of a regular chow (TRF-Reg) diet. We adapted an automated feeder system to dispense 300 mg pellets of either CR or regular chow at even intervals throughout the entire dark period (Fig. 1a). Each night, TRF-Reg mice received a pellet of regular chow (Bio-Serv F06381, ‘AIN-93M’) every 1.2 h throughout the dark period (for a total of 3 g), while TRF-CR mice received a pellet of a carbohydrate-depleted, but protein-, fat- and micronutrient-controlled chow (Bio-Serv F07391, ‘AIN-93M 40% DR’) every 2 h throughout the dark period (for a total of 1.8 g), resulting in a 40% reduction in calorie intake from carbohydrate (Fig. 1a and Supplementary Table 1). Importantly, this feeding protocol did not induce differences in wheel-running behaviour, daily activity onset or molecular rhythms (PER2::LUC) of the central circadian pacemaker in the suprachiasmatic nucleus (SCN) of the hypothalamus in TRF-CR mice compared with TRF-Reg controls (Extended Data Fig. 1a), thus circumventing food entrainment commonly caused by CR regimens in which food restriction for brief windows of time disrupts the endogenous rest/activity cycle^18^. We examined mice after 4 weeks, when mice fed TRF-CR reached their minimum weight and displayed improved glucose tolerance and increased hepatic gluconeogenesis from lactate, consistent with published phenotypes of CR (Extended Data Fig. 1b–d)^6,19^. Under this model, we performed semiquantitative high-performance liquid chromatography (HPLC) analysis of separate basic and acidic extractions to measure the concentrations of total NADH and NAD^+^, respectively, in liver during the day and night^20,21^, because accurate free versus bound hepatic NADH measurements are not possible. We observed that total NADH levels were approximately threefold higher in livers of TRF-CR compared with TRF-Reg mice during the daytime (zeitgeber time (ZT) 4) (Fig. 1b), whereas NADH levels were slightly reduced at night (ZT16) (Extended Data Fig. 1e). Because ad libitum NADH levels peak at ~40 pmol per mg liver during the daytime (Extended Data Fig. 1e), we estimate daytime total NADH levels during TRF-CR are ~120 pmol per mg liver (~0.12 mM given a density of 1 g ml^−1^ for liver). This approximately threefold increase in daytime NADH levels following CR is consistent with previous reports in liver showing a 2.4-fold increase in NADH from ~140 to 337 pmol per mg liver (0.14–0.337 mM)^22^. Because NADH levels peak during the day and trough at night (Extended Data Fig. 1e), our results suggest that TRF-CR amplifies daily NADH rhythms in liver. Interestingly, we did not observe significant changes in hepatic NAD^+^ levels by TRF-CR in either the day or night (Fig. 1b and Extended Data Fig. 1f), similar to previous reports of total cell daytime hepatic NAD^+^ levels not changing during CR^22^, suggesting that NAD^+^ rhythms, which typically peak during the dark period, were unaffected by TRF-CR in liver.

Liver is a key metabolic organ that governs whole-body metabolism during fasting and low-nutrient conditions by releasing metabolites into the circulation that can be oxidized by other tissues, including brain and brown adipose tissue (BAT)^23^. For example, the partial oxidation of fatty acids (derived from white adipose tissue during fasting/sleep) in the liver produces acyl-carnitines that are secreted into serum and serve as a thermogenic fuel source for BAT^24–26^. To determine whether TRF-CR alters hepatic mobilization of metabolite stores during the daytime, we first profiled acyl-carnitines in both liver and serum from TRF-CR mice using tandem mass spectrometry (MS/MS), as described previously^27,28^. TRF-CR decreased acyl-carnitine species of all lengths in serum (Fig. 1c), and medium- and long-chain acyl-carnitine species in liver (Extended Data Fig. 1g), suggesting that hepatic production of acyl-carnitines was reduced with TRF-CR. Because hepatic acyl-carnitine production has been linked to regulation of body temperature at the level of BAT^24^, we next examined body temperature rhythms by implanting temperature transponders into freely moving mice during TRF-CR. As has been reported, mice on regular chow diet demonstrate a circadian variation in body temperature (Fig. 1d)^29,30^. TRF-CR led to a daily rhythmic reduction in core body temperature relative to TRF-Reg mice, predominantly during the early daytime hours (Fig. 1d), consistent with previous reports in one-meal CR paradigms^5,31^. We note that the body temperature of TRF-CR mice is not directly dependent upon the availability of food pellets, because body temperature begins to: (1) decrease in the late night-time before access to the last pellet of food (at ZT22); and (2) normalize at midday (at ZT8), 4 h before refeeding, when animals are still fasting. Together, these results reveal that TRF-CR leads to elevation of hepatic NADH content concurrent with reduced acyl-carnitine production and the nadir in daily core body temperature rhythms during CR.

To determine whether the increase in NADH per se inhibits﻿ hepatic lipid oxidation during TRF-CR, we took advantage of a novel genetic tool recently developed to manipulate NADH levels without changing the nutrient state of the animal using flavin adenine dinucleotide-dependent *Lb*NOX, which reduces oxygen to water and has specificity for oxidizing NADH over NADPH (Fig. 1e)^16,17^. Although NADH exists in distinct mitochondrial and cytonuclear pools, we chose to utilize the cytonuclear form of *Lb*NOX to manipulate NADH levels because the increased lactate flux into gluconeogenesis during TRF-CR (Extended Data Fig. 1d) suggests a net increase in cytosolic NADH due to the export of NADH equivalents from the mitochondria into the cytonuclear compartment via the malate–aspartate shuttle^32–35^, although accurate subcellular quantification is not possible in liver. Before subjecting mice to TRF-CR, we transduced cytonuclear *Lb*NOX under a liver-specific promoter to wild-type mice by retro-orbital injection of adeno-associated viruses (AAVs) that expressed either *Lb*NOX or an empty vector (‘null’). We observed liver-specific *Lb*NOX expression (Fig. 1e) and reduced levels of daytime NADH during TRF-CR without changes in the levels of oxidized NAD^+^ (Fig. 1f). To determine whether the altered acyl-carnitine profiles and whole-body temperature rhythms during TRF-CR depend on elevated hepatic NADH during TRF-CR, we assessed acyl-carnitine levels in liver and serum from *Lb*NOX-expressing TRF-CR-fed mice following monitoring of body temperature rhythms from implanted temperature transponders. Remarkably, we observed that hepatic *Lb*NOX overexpression resulted in increased acyl-carnitine levels in both liver (Extended Data Fig. 1h) and serum (Fig. 1g) during TRF-CR and abrogated the daily reduction in core body temperature characteristic of the TRF-CR mice (Fig. 1h). These results indicate that the elevated levels of cytonuclear NADH in the liver during daytime both inhibits acyl-carnitine production and decreases core body temperature during TRF-CR.

### NADH regulates metabolic transcription factors during TRF-CR

**Fig. 2:**
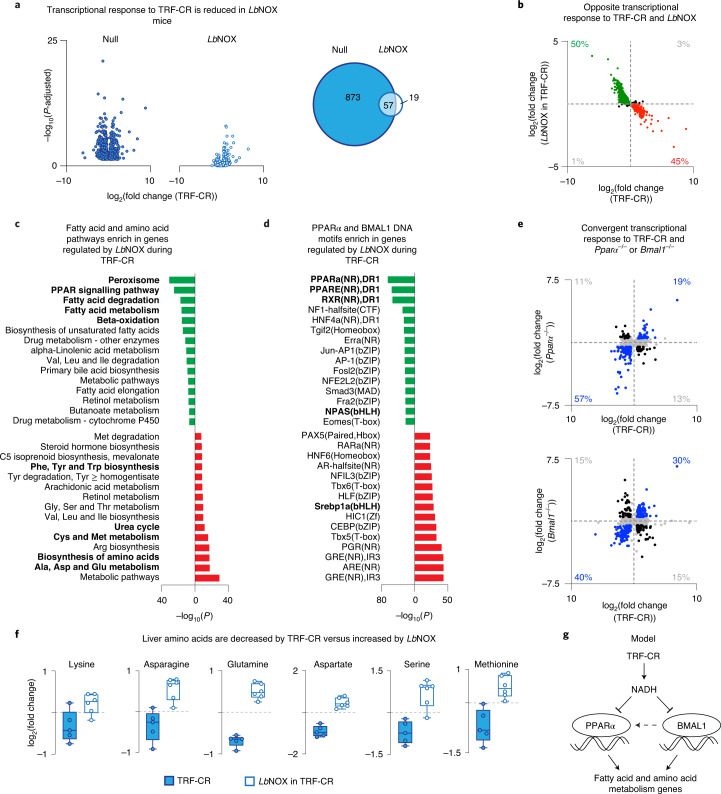
Daytime NADH elevation regulates genome-wide transcription of fatty acid and amino acid metabolism genes during TRF-CR. **a**, RNA-Seq in liver in the morning (ZT4) demonstrating the effect of TRF-CR in null-transduced (left) (*n* = 6) and *Lb*NOX-transduced (middle) (*n* = 6 for TRF-Reg, *n* = 5 for TRF-CR) 4–6-month-old male C57BL/6J mice for genes differentially expressed (DESeq2 FDR-adjusted *P* < 0.05) by TRF-CR in null-transduced mice (930 genes). Venn diagram (right) displays overlap in differentially expressed genes by TRF-CR in null- and *Lb*NOX-transduced mice. **b**, Quadrant plot comparing transcriptional responses between TRF-CR (*x* axis) and *Lb*NOX in TRF-CR (*y* axis). Each point indicates a gene that is differentially expressed by TRF-CR in null-transduced mice (930 genes). Colouring indicates genes within quadrant 2 (green) and quadrant 4 (red), and the percentages within each quadrant are shown. **c**,**d**, For the genes within quadrants 2 and 4 from **b**, the top 15 (**c**) Kyoto Encyclopedia of Genes and Genomes (KEGG) terms enriched (*P* < 0.05) following gene ontology analysis and (**d**) JASPAR motifs enriched (*P* < 0.05) following HOMER DNA motif enrichment analysis are shown. **e**, Quadrant plots comparing the transcriptional response to TRF-CR in null-transduced mice (*x* axis) with that of genetic ablation of either *Pparα* (top) or *Bmal1* (bottom) (*y* axis*)* in animals fed ad libitum. Each﻿ point indicates a gene that is differentially expressed by TRF-CR in null-transduced animals. Genes that have an absolute log_2_(fold change) > 0.5 for both comparisons are coloured blue or black, and the percentage of genes was determined by quadrant (*n* = 3). **f**, LC–MS metabolomics profiling of amino acids in liver during the daytime (ZT4). The log_2_(fold change) from TRF-CR (blue) (*n* = 5) and *Lb*NOX in TRF-CR (white) (*n* = 6) is shown for select differential amino acids (two-tailed, unpaired Student’s *t* test with Benjamini and Hochberg adjustment for multiple measures FDR *P* < 0.05; see Supplementary Table 2 for full list of amino acids). Box and whisker plots depict the following: line, median; box limits, first and third quartiles; whiskers, 10th and 90th percentiles. **g**, Model depicting the interrelationship of NADH during TRF-CR to the activity of PPARα and BMAL1 and the transcription of downstream oxidative gene networks﻿.

**Extended Data Fig. 2:**
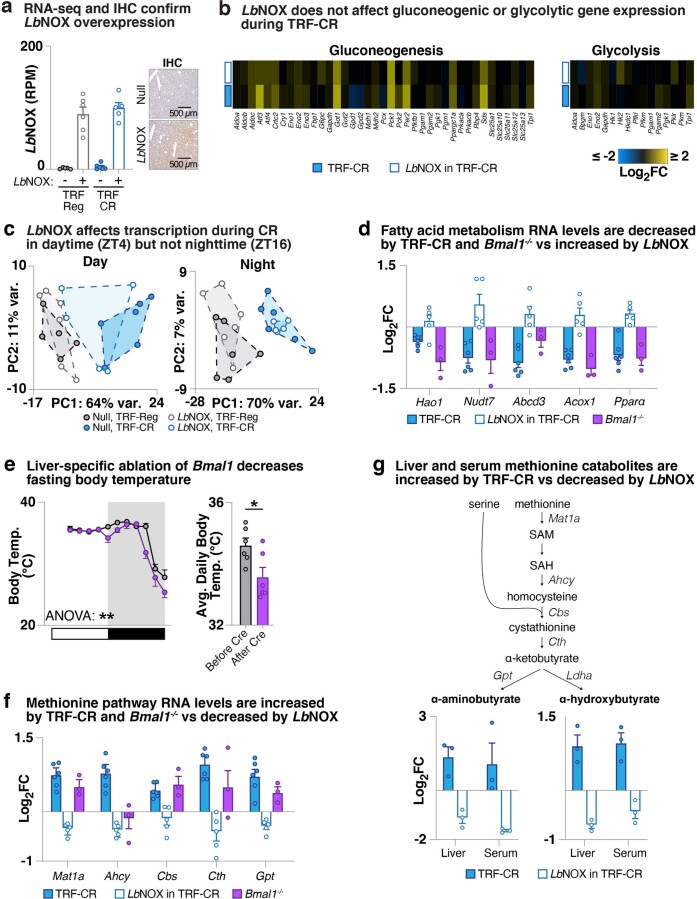
Daytime NADH elevation regulates genome-wide transcription of fatty acid metabolism genes through BMAL1 during TRF-CR. **a**, (*left*) RNA-seq reads per 10 million sequenced reads (RPM) that align to *Lb*NOX in *Lb*NOX- vs null-transduced TRF-Reg (n = 6 per genotype) and TRF-CR (n = 6 for null; n = 5 for *Lb*NOX) liver. (*right*) Representative *in situ* immunohistochemistry against FLAG-*Lb*NOX in liver of null- and *Lb*NOX-transduced mice. Experiments were performed in 4–6 mo old male C57BL6/J mice unless otherwise noted. **b**, Heatmap depicting log_2_-FC in gene expression from TRF-CR and *Lb*NOX in TRF-CR for genes within gluconeogenic and glycolytic gene ontology groups. **c**, Unbiased principal components analysis during the day (ZT4) or night (ZT16) of null- and *Lb*NOX-expressing TRF-Reg or TRF-CR mice for genes that are differentially-expressed by TRF-CR in null mice at respective time points (DESeq2 p-adj<0.05) (n as in panel a). **d**, Log_2_-transformed FC in expression of select fatty acid metabolism genes during TRF-CR (blue) (n = 6), following *Lb*NOX-overexpression during TRF-CR (white) (n = 5), and in *Bmal1* knockout liver (purple) (n = 3). **e**, 24-hour fasted body temperatures in 4–6 mo old female liver-specific *Bmal1*^*fx/fx*^ mice before and after retro-orbital administration of AAV8–TBG-iCre (n = 6) (*left*: ANOVA, p < 0.01; *right:* *p < 0.05 in paired, two-way student’s t test). **f**, Log_2_-transformed FC in expression of genes within the methionine pathway for conditions (n as in panel d). **g**, (*top*) Summary of methionine pathway and (*bottom*) metabolomics of effect of TRF-CR and *Lb*NOX in TRF-CR on end-products of methionine metabolism in liver and serum performed during the daytime (ZT4) (n = 3). Data are presented as mean values ± SEM.

NAD^+^ supplementation increases core clock transcriptional activity and reprogrammes genome-wide transcription of oxidative genes collaboratively controlled through BMAL1–PPARα^10,36–38^. Further, in yeast, the transcriptional response to CR requires rate-limiting enzymes in NAD^+^ biosynthesis^4^. Thus, we next sought to understand whether the changes in NADH that occur in the cytonuclear compartment of mouse liver during TRF-CR affect genome-wide transcription of metabolic and oxidative gene networks. We performed RNA sequencing (RNA-Seq) in liver of null- and *Lb*NOX-transduced TRF-CR and TRF-Reg mice during the daytime (Fig. 2a and Extended Data Fig. 2a). We identified 930 genes differentially expressed by TRF-CR versus TRF-Reg (DESeq2, false discovery rate (FDR)-adjusted *P* < 0.05) (Fig. 2a, left). Of those 930 differentially expressed genes, only 57 were still differentially expressed by TRF-CR in the presence of *Lb*NOX (Fig. 2a, middle), whereas 19 genes were uniquely differentially expressed by TRF-CR in the presence of *Lb*NOX (Fig. 2a, right). We next examined the effect of *Lb*NOX on the expression of the 930 genes regulated by TRF-CR. We observed that *Lb*NOX expression in TRF-CR mice caused opposite changes in expression relative to TRF-CR for 95% of genes (Fig. 2b) (binomial test *P* < 2.2 × 10^−16^). These observations indicate that reduction of NADH levels by *Lb*NOX counteracts the transcriptional response to TRF-CR for a majority of genes that are differentially expressed by TRF-CR during the daytime. Of note, principal component analysis revealed that *Lb*NOX did not affect gene expression in the night (ZT16) (Extended Data Fig. 2c), suggesting that *Lb*NOX regulates the transcriptomic response to TRF-CR specifically in the daytime (ZT4) when NADH is elevated.

We next applied unbiased in silico transcriptomic and motif analyses to identify the gene networks and TFs that were altered by *Lb*NOX during TRF-CR. Using gene ontology analysis, we found that genes that were decreased by TRF-CR and increased by *Lb*NOX (green—‘group 1’) were enriched in gene ontologies associated with fatty acid oxidation and PPARα signalling, whereas genes that were increased during TRF-CR and decreased by *Lb*NOX (red—‘group 2’) were enriched in gene ontologies associated with amino acid metabolism (Fig. 2c). We note that although we found gene ontologies associated with fatty acid and amino acid metabolism, we did not observe enrichment of gene ontologies associated with either gluconeogenesis or glycolysis (Extended Data Fig. 2b). The discovery of gene ontologies associated with fatty acid metabolism is consistent with our finding that TRF-CR decreases acyl-carnitine levels through a mechanism that is reversed by *Lb*NOX (Fig. 1, Extended Data Fig. 1).

Using DNA motif analysis, we next found that canonical PPARα-regulated genes were specifically enriched among those genes that were upregulated in *Lb*NOX-expressing TRF-CR mice (group 1 above), whereas we found enrichment of the bHLH DNA motif that is recognized by CLOCK/BMAL1 among genes that were either up- or downregulated by *Lb*NOX in TRF-CR mice (groups 1 and 2) (Fig. 2d). Further, the hepatic transcriptome pattern of mice subjected to TRF-CR has a strong positive correlation with the hepatic gene expression profile of *Pparα* nullizygous mice^39^ (Fig. 2e, top) (log_2_(fold change) cut-off ± 0.5; 76% of genes, binomial test *P* < 7.346 × 10^−16^). The observation that patterns of NADH-sensitive gene expression in liver of TRF-CR mice overlap with that of PPARα-null animals indicates a role for redox state in the transcription control of acyl-carnitine production and thermogenesis^24,37,40,41^.

Interestingly, we observed that *Bmal1* ablation is sufficient to decrease expression of *Pparα* and other fatty acid oxidation genes that are decreased by TRF-CR and increased by *Lb*NOX (Extended Data Fig. 2d), consistent with previous findings that BMAL1 is a positive regulator of fatty acid oxidation and PPARα expression^36,38,42,43^. Comparison of the genome-wide expression profile of TRF-CR with that of *Bmal1* ablation (GSE133989)^10^ revealed a strong positive correlation in gene expression between these conditions (log_2_(fold change) cut-off ±0.5; 70% of genes, binomial test *P* < 1.17 × 10^−^^13^) (Fig. 2e, bottom). To test whether hepatic BMAL1 also plays a role in the thermogenic response to low-nutrient conditions, we measured body temperature in mice harbouring a floxed allele of *Bmal1* before and after administration of AAV8-thyroid binding globulin-improved Cre recombinase (AAV8–TBG–iCre) to specifically delete *Bmal1* in the liver. When mice were fasted at the start of the light period (ZT0) to induce a low-energy state, we found that liver-specific *Bmal1* knockout animals displayed decreased body temperature (Extended Data Fig. 2e).

### Hepatic NADH controls amino acid metabolism during TRF-CR

Because gene networks involved in amino acid metabolism were increased in expression by TRF-CR and decreased by *Lb*NOX (Fig. 2c, red), we next measured concentrations of amino acid and hydrophilic metabolites using liquid chromatography–mass spectroscopy (LC–MS) in liver isolated from null- or *Lb*NOX-transduced mice maintained on TRF-Reg and TRF-CR diets. We observed that amino acids involved in nitrogen metabolism (including lysine, asparagine, glutamine and aspartate) were decreased by TRF-CR and increased by *Lb*NOX (Fig. 2f and Supplementary Table 2) (multiple *t* test, FDR-adjusted *P* < 0.05). This finding is consistent with enrichment of gene ontology pathways associated with the urea cycle, which sequesters nitrogen (Fig. 2c). Interestingly, we also observed that oxygen/sulfur-containing amino acids such as serine and methionine were decreased by TRF-CR and increased by *Lb*NOX (Fig. 2f and Supplementary Table 2) (multiple *t* test, FDR-adjusted *P* < 0.05), consistent with the enrichment of gene ontology terms associated with cysteine and methionine metabolism (Fig. 2c). Specifically, we observed increased expression of RNAs encoding enzymes within the methionine metabolic pathway, such as *Mat1a*, *Ahcy*, *Cbs* and *Cth*, in TRF-CR mice, and decreased expression of these genes in *Lb*NOX-expressing mice (Extended Data Fig. 2f). We similarly observed that *Bma1l* nullizygous animals exhibited increased expression of *Mat1a*, *Cbs* and *Cth* (Extended Data Fig. 2f). To interrogate the extent to which metabolites downstream of methionine and serine are regulated by NADH during TRF-CR, we quantified metabolites by LC–MS in liver and serum from null virus and *Lb*NOX TRF-CR mice. Consistent with the transcriptional analyses, our metabolomic studies revealed that catabolites within the methionine/serine pathway, including α-aminobutyrate and α-hydroxybutyrate, were increased by TRF-CR and decreased by *Lb*NOX in TRF-CR (Extended Data Fig. 2g). We note that within the methionine metabolic pathway, the conversion of α-ketobutyrate into α-hydroxybutyrate by LDHA is the only biochemical reaction that utilizes NAD(H) as a cofactor (Extended Data Fig. 2g). These results reveal that NADH redox state during daytime controls both methionine and serine catabolism in response to TRF-CR. Together, these data suggest that elevated daytime NADH during TRF-CR inactivates BMAL1 and PPARα in the liver, causing decreased expression of fatty acid oxidation genes, increased expression of genes involved in amino acid metabolism and reduced core body temperature (Fig. 2g).

### NADH inhibits SIRT1 to link energy state to transcription

**Fig. 3:**
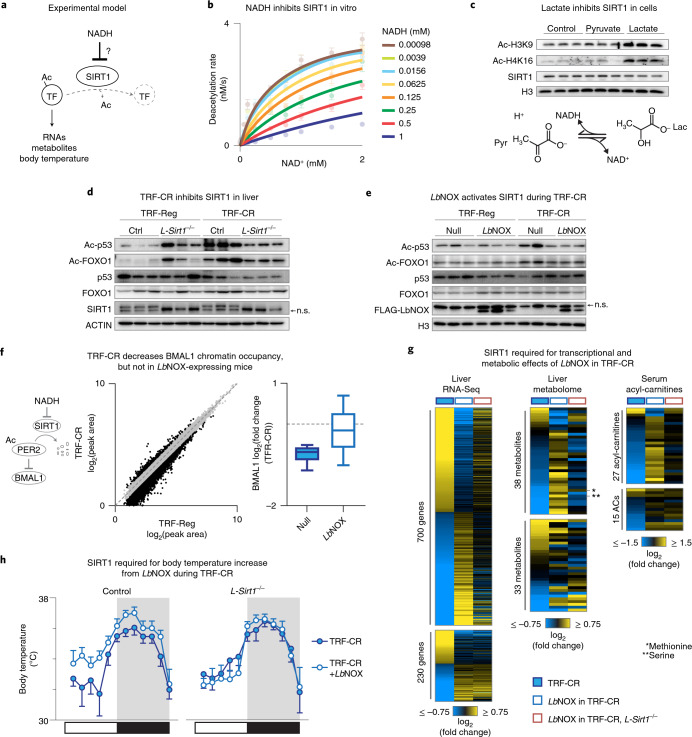
NADH inhibits SIRT1 in the morning during TRF-CR to regulate metabolism and body temperature. **a**, Model to examine the role of SIRT1 in the NADH-dependent effects on RNAs, metabolites and body temperature during TRF-CR. **b**, Deacetylation rate for SIRT1 with increasing concentrations of NAD^+^ and NADH assayed by SAMDI-MS (*n* = 4). **c**, Model of pyruvate/lactate equilibrium shows that supplementation with pyruvate and lactate reduces and elevates NADH, respectively. Western blotting for SIRT1 targets, Ac-H3K9 and Ac-H4K16, in immortalized mouse embryonic fibroblasts treated with pyruvate or lactate to modulate NADH (*n* = 3). **d**,**e**, Western blotting for SIRT1 targets, Ac-p53 and Ac-FOXO1, in TRF-Reg or TRF-CR liver of 4–6-month-old male (**d**) control or liver-specific *Sirt1*^−*/*−^ mice or (**e**) null- or *Lb*NOX-transduced mice. n.s., non-specific. Uncropped western blot scans labelled with molecular weight markers are presented in the Source Data files. **f**, BMAL1 ChIP-Seq in liver of TRF-Reg or TRF-CR mice. Peaks demonstrating an absolute log_2_(fold change) > 0.5 are coloured black. Box–whisker plots of BMAL1 ChIP-Seq demonstrating the effect of TRF-CR in null- (*n* = 6) and *Lb*NOX-transduced (*n* = 3) liver on BMAL1 peaks identified in controls and with an absolute log_2_(fold change) > 0.5 in controls. Box and whisker plots depict the following: line, median; box limits, first and third quartiles; whiskers, 10th and 90th percentiles. **g**, Heatmap depicting log_2_(fold change) in gene expression (left), liver metabolite concentrations (middle) and serum acyl-carnitine levels (right) in indicated conditions/genotypes at ZT4. Heatmaps are subdivided into the genes, metabolites and acyl-carnitines that are regulated by *Lb*NOX during TRF-CR through mechanisms requiring SIRT1 and sorted by effect of TRF-CR (RNA-Seq, *n* = 6; metabolomes, *n* = 5–6; acyl-carnitines *n* = 3). **h**, Body temperature rhythms over 24 h from subcutaneous probes implanted in null- (*n* = 6 for control, *n* = 5 for *L-Sirt1*^−*/*−^) or *Lb*NOX-expressing (*n* = 5) control and liver-specific *Sirt1*^−*/*−^ mice on TRF-CR. Data are presented as mean﻿ values ± s.e.m. Source data

**Extended Data Fig. 3:**
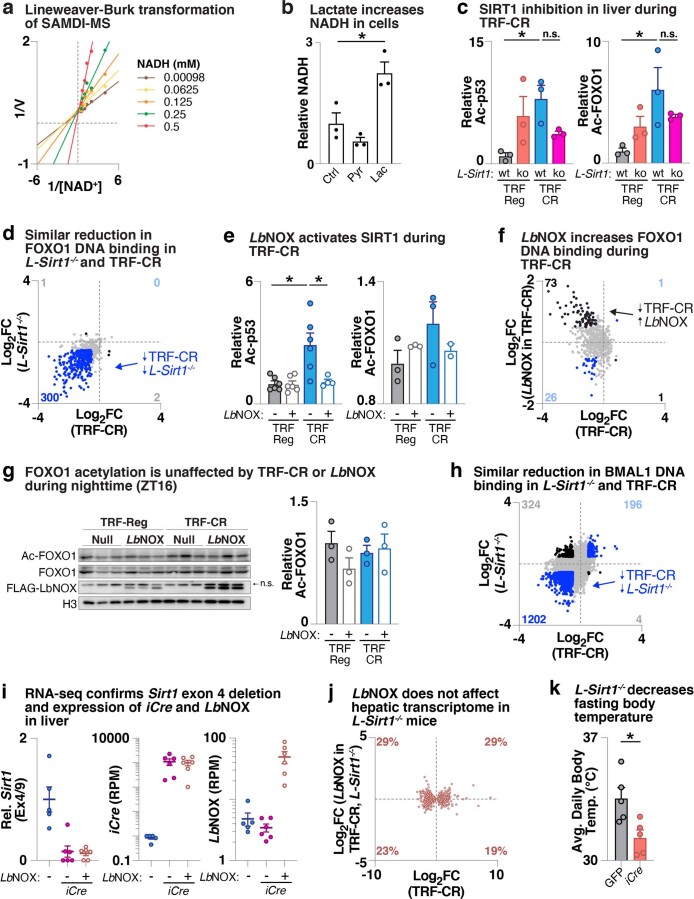
NADH inhibition of SIRT1 activity. **a**, Lineweaver-Burk transformation of data from SAMDI-MS. **b**, Relative NADH quantified by HPLC following supplementation with pyruvate or lactate compared to controls (n = 3). **c**, Densitometric quantification of western blots from liver of 4–6 mo old male mice in indicated genotypes at ZT4 for p53-(K379)Ac (*left*) and FOXO1-(K242, K245, K262)Ac (*right*) relative to control TRF-Reg (from Fig. 3d) (n = 3). **d**, FOXO1 ChIP-seq from liver collected during the daytime comparing effect of FOXO1 binding in TRF-CR (x-axis) with the effect of hepatic SIRT1 ablation (*L-Sirt1*^−*/*−^) (y-axis). Each point indicates a FOXO1 peak in control liver. Peaks that have absolute log_2_(fold change) >0.5 for both comparisons are colored blue or black and counted by quadrant (n = 2). **e**, Densitometric quantification of western blots as in (c) relative to null-transduced TRF-Reg mice (from Fig. 3e) (Ac-p53: n = 4 for TRF-CR, *Lb*NOX. n = 6 for all other conditions; Ac-FOXO1: n = 2 for TRF-CR, *Lb*NOX. n = 3 for all other conditions). **f**, FOXO1 ChIP-seq from liver during the daytime comparing effect of TRF-CR (x-axis) (n = 3) with effect of (f) *Lb*NOX in TRF-CR mice (y-axis) (n = 2) with coloring and counting as in panel d. (n = 2–3). **g**, Western blotting for Ac-FOXO1 during the nighttime (ZT16) in null- and *Lb*NOX-transduced mice on TRF-Reg and TRF-CR (n.s. non-specific) and densitometric quantification (n = 3). **h**, BMAL1 ChIP-seq from liver during the daytime comparing the effect of TRF-CR and *L-Sirt1*^−*/*−^ as in panel d (n = 3). Uncropped Western blot scans labelled with molecular weight markers are presented in the Source Data Files. **i**, RNA-seq reads mapping to the exon of *Sirt1* that is flanked by *LoxP* sites (Ex4) relative to exon 9 (Ex9) of *Sirt1*, and RNA-seq reads per 10 million sequenced reads (RPM) that align to iCre and *Lb*NOX in null- or *Lb*NOX-transduced TRF-CR mice co-transduced with iCre (n = 6). Null-expressing mice on TRF-CR (blue) (n = 5) are shown as reference. **j**, Quadrant plot comparing transcriptional responses to TRF-CR in null-transduced mice (x-axis) (n = 6) and *Lb*NOX-expression in TRF-CR, *L-Sirt1*^−*/*−^ mice (y-axis) (n = 6). Each point indicates a gene that is DE by TRF-CR in null-transduced mice (930 genes). Percentages of genes within each quadrant are shown. **k**, Average 48-hour fasting body temperature in 4–6 mo old female liver-specific *Sirt1*^−*/*−^ mice (n = 5). Data are presented as mean values ± SEM. Statistics were performed with unpaired, two-tailed student’s t test except as otherwise noted in the figure. *p < 0.05. Source data

Our data suggest that elevated daytime NADH during TRF-CR inactivates BMAL1 and PPARα in the liver (Figs. 1 and 2). Because nutrient-dependent deacetylase SIRT1 regulates the activity of both BMAL1 and PPARα^10,11,37,44^, we hypothesized that the elevated NADH during TRF-CR may inactivate BMAL1 and PPARα through SIRT1 (Fig. 3a). The relationship between NADH levels and sirtuin activity in response to time-restricted hypocaloric feeding remains uncertain in mammals, with inconsistent findings from prior biochemical analyses concerning the role of NADH due to differences across isoforms, species and assay conditions^45–47^. We therefore sought to re-evaluate SIRT1 activity under physiological range concentrations of both NAD^+^ and NADH by using a direct MS method, self-assembled monolayers for matrix-assisted laser desorption/ionization (SAMDI-MS)^48^, which circumvents problems associated with fluorescence-based assays of deacetylase function^49–51^. Recombinant SIRT1 was incubated with acetyl-peptide substrate (GRK^Ac^RVC) in the presence of varying concentrations of NAD^+^ (0–2 mM) and NADH (0–1 mM), followed by quenching and immobilization of the acetylated and deacetylated peptides on a maleimide-functionalized gold surface and quantification of the deacetylation rate by MS. Whereas NAD^+^ stimulated SIRT1 deacetylase activity as expected, increasing concentrations of NADH inhibited the activity of SIRT1 (Fig. 3b and Extended Data Fig. 3a). Importantly, the inhibition constant K_i__(NADH)_ value for SIRT1 that we observed (0.124 mM) falls within previously reported ranges for physiological concentrations of total NADH in mouse liver (100–300 pmol per mg liver, or 0.1–0.3 mM)^22,52–54^ and is within range of our semiquantitative determination of total NADH levels during TRF-CR (~120 pmol per mg liver, or ~0.12 mM), estimated based on a threefold increase in daytime NADH levels relative to mice fed ad libitum (Extended Data Fig. 1e). Thus, SIRT1 is inhibited by NADH at concentrations that have been reported for total NADH in the whole liver, although accurate determination of NADH in the nucleus of liver is not currently possible.

To test whether NADH inhibits SIRT1 in vivo, we first supplemented mouse embryonic fibroblasts with lactate (to increase NADH) or pyruvate (to decrease NADH) and analysed the acetylation state of the targets of SIRT1 by immunoblotting (Ac-H3K9, Ac-H4K16) (Extended Data Fig. 3b,c)^55,56^. Acetylated H3K9 and H4K16 increased following lactate supplementation (Fig. 3c), suggesting reduced SIRT1 activity. To examine whether elevated daytime NADH levels during TRF-CR led to altered SIRT1 activity in liver, we analysed the lysine acetylation of two well-characterized SIRT1 targets, p53 (on K379) and FOXO1 (on K242, K245 and K262)^57–60^. We observed increased lysine acetylation of both targets in TRF-CR compared with TRF-Reg mice during the daytime, similar to hyperacetylation of these TFs in liver-specific *Sirt1* knockout mice (*L-Sirt1*^−*/*−^) (Fig. 3d and Extended Data Fig. 3c). Further, recruitment of FOXO1 to DNA was reduced during the day in TRF-CR mice, similar to the decreased genome-wide FOXO1 binding observed in *L-Sirt1*^−*/*−^ liver (Extended Data Fig. 3d)^59,60^. Transduction of cytonuclear *Lb*NOX in the liver during TRF-CR reduced FOXO1 and p53 acetylation during the daytime but not the night-time, consistent with daytime activation of SIRT1 by *Lb*NOX (Fig. 3e and Extended Data Fig. 3e,g). Finally, we observed increased DNA binding of FOXO1 in *Lb*NOX-treated versus null-transduced TRF-CR animals during the daytime (Extended Data Fig. 3f). These results reveal that hepatic SIRT1 activity is reduced by NADH in the daytime during TRF-CR.

SIRT1 has been shown to increase chromatin occupancy of the circadian clock activator complex^10^, leading us to analyse whether the increase in morning levels of NADH may in turn reduce DNA binding of BMAL1. Here, we observed reduced BMAL1 chromatin occupancy in TRF-CR mice by chromatin immunoprecipitationsequencing (ChIP-Seq) (Fig. 3f), similar to the reduction in BMAL1 chromatin occupancy observed in liver-specific *Sirt1* knockout mice (Extended Data Fig. 3h) (*P* < 6.4 × 10^−^^11^)^10^. To assess the impact of lowering NADH levels on BMAL1 recruitment to chromatin during TRF-CR, we performed BMAL1 ChIP-Seq in *Lb*NOX-expressing TRF-Reg and TRF-CR mice. We observed that of the 6,042 BMAL1 peaks that were downregulated in TRF-CR mice; only 1,880 (31%) were also downregulated from TRF-CR in *Lb*NOX mice (Fig. 3f, right) (log_2_(fold change) < −0.5, binomial test *Lb*NOX: *P* < 2.2 × 10^−^^16^). Thus, NADH and SIRT1 participate in the control of BMAL1 chromatin binding during TRF-CR.

Based upon the observations that NADH inhibits SIRT1, and that NADH levels mediate the daytime transcriptional and metabolic response to TRF-CR (Fig. 2), we next sought to determine whether *Lb*NOX alters the response to TRF-CR via a mechanism requiring activation of SIRT1. To test whether NADH oxidation reprogrammes transcription via SIRT1, we first performed RNA-Seq to examine the effect of *Lb*NOX in liver-specific *Sirt1* knockout animals subjected to TRF-CR. *Sirt1* floxed animals were cotransduced with AAV8–TBG–iCre and AAVs expressing either *Lb*NOX or the null vector before initiation of TRF-CR (Extended Data Fig. 3i). Whereas *Lb*NOX expression opposed the transcriptional response to TRF-CR in control animals (Figs. 2b and 3g, left), we observed that *L-Sirt1* knockout abrogated the response to *Lb*NOX for 700 of 930 TRF-CR genes (Fig. 3g, left, Extended Data Fig. 3j and Supplementary Table 3). We next tested whether SIRT1 was necessary for the effects of *Lb*NOX on amino acid and metabolite concentrations during TRF-CR by performing unbiased metabolomics in the same mice as above. Whereas *Lb*NOX expression increased the concentration of amino acids in control animals during TRF-CR (Fig. 2f) and generally counteracted the effect of TRF-CR on metabolite concentrations (Fig. 3g, middle), we found that *L-Sirt1* knockout abrogates the effect of *Lb*NOX on the majority (38 of 71) of hepatic metabolites (Fig. 3g, middle, and Supplementary Table 3). Finally, we determined whether SIRT1 is required for the increase in serum acyl-carnitine levels by *Lb*NOX during TRF-CR by profiling acyl-carnitine concentrations in the serum of the same mice as above. Whereas *Lb*NOX increases acyl-carnitines when expressed in control animals (Figs. 1g and 3g, right), 27 of 42 acyl-carnitines were unaffected by *Lb*NOX when it was expressed in *L-Sirt1* knockout animals (Fig. 3g, right, and Supplementary Table 3). These results show that the transcriptional and metabolic response to *Lb*NOX during TRF-CR requires SIRT1.

Changes in hepatic metabolism led to whole-animal energy conservation during the daytime under TRF-CR; therefore, we next sought to investigate the extent to which SIRT1 was required for *Lb*NOX to increase body temperature during the daytime in response to TRF-CR. First, we assessed fasting body temperature in mice in which we had ablated *Sirt1* in the liver by transduction of AAV-iCre, and observed reduced fasting body temperature (Extended Data Fig. 3k). Next, we determined the extent to which SIRT1 is necessary for increased core body temperature in *Lb*NOX-expressing mice subjected to TRF-CR. Here, we generated animals that express *Lb*NOX and have *L-Sirt1* knockout by cotransducing *Sirt1* floxed animals with AAVs expressing *Lb*NOX, iCre or control vectors, then maintained them on TRF-CR for 4 weeks before measuring body temperature. We observed an increase in body temperature from *Lb*NOX in mice expressing intact SIRT1 (Fig. 3h, left). Remarkably, mice in which *L-Sirt1* was ablated had no change in body temperature from *Lb*NOX during TRF-CR (Fig. 3h, right). We also note that both control mice subjected to TRF-CR and *L-Sirt1* knockout mice on TRF-CR have similar body temperatures, suggesting that decreased activity of SIRT1 results in lower core body temperature under TRF-CR. Together, these findings show that hepatic NADH drives rhythmic energy conservation during TRF-CR by inhibiting SIRT1.

## Discussion

**Fig. 4:**
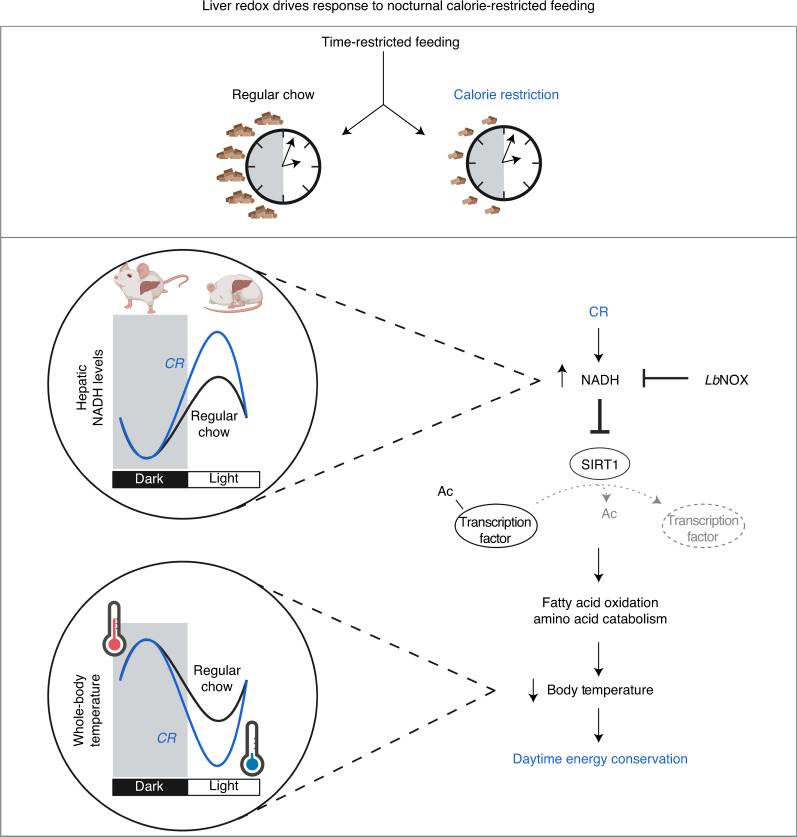
*Lb*NOX redox state in liver drives energy conservation during nocturnal CR feeding. TRF-CR generates rhythmic bouts of daytime torpor through increased levels of NADH in liver, inhibition of SIRT1 and downregulation of oxidative gene networks controlling acyl-carnitines and core body temperature. Reducing levels of NADH in the morning through the transduction of *Lb*NOX in TRF-CR mice increases lipid oxidation through the activation of SIRT1, resulting in elevated daytime body temperature rhythms. These findings identify NAD(H) redox state as a link between TRF-CR and whole-body metabolism﻿.

Results presented here reveal that the circadian system participates in the response to a low-energy state associated with hypocaloric feeding through the rhythmic elevation of NADH during the daytime and inhibition of SIRT1 in liver. In nocturnal rodents provided a CR diet limited to the active period, we observed that daytime NADH elevation drives a daily drop in core body temperature in the morning, rhythmic inhibition of SIRT1, decreased BMAL1-dependent gene expression and reduced acyl-carnitine and amino acid levels. We speculate that NADH regulates energy conservation in the morning when food becomes scarce for nocturnal mice through the inhibition of SIRT1-controlled gene networks (Fig. 4).

Overexpression of the cytonuclear NADH-specific oxidase *Lb*NOX, in combination with liver-specific ablation of *Sirt1*, has enabled us to dissociate hepatic redox and nutritional state, providing insight into how NADH controls SIRT1 in vivo during the daily fasting period^16^. We find that alleviation of NADH inhibition of SIRT1 during the daytime leads to genome-wide disinhibition of SIRT1-controlled RNAs during TRF-CR. In addition, liver isolated from *Lb*NOX-transduced animals was enriched in PPARα-regulated transcripts, a major transcription factor involved in the switch to oxidative metabolism during fasting^41^. It is likely that downregulation of PPARα and BMAL1 in the morning/fasting period during TRF-CR contributes to the daily rhythms in energy conservation characterized by reduced body temperature and entry into torpor during the fasting period^5,31^. Indeed, downregulation of hepatic fatty acid oxidation has recently been shown to exacerbate cold sensitivity^24^. Finally, identification of the methionine pathway downstream of NADH/SIRT1/BMAL1 during TRF-CR raises the possibility that the healthful effects of both calorie and methionine restriction may be mediated through a common mechanism^61–63^. Strategies to mimic intermittent NADH redox inhibition of SIRT1 in liver may recapitulate beneficial effects observed with TRF and be leveraged towards altering fuel selection in cell types involved in proliferative and inflammatory disease states.

Although sirtuins have been associated with the beneficial effects of CR across a range of organisms^7,22,64,65^, and CR promotes longevity in yeast by increasing ySir2p activity and NAD^+^/NADH^4,45^, the situation is more complex in mammals, because CR exerts tissue-specific effects on NAD^+^ and NADH^22,66,67^. Our findings using SAMDI-MS indicate that NADH inhibits SIRT1 activity at physiologically relevant concentrations^22,52–54^, and our in vivo genetic analyses indicate that hepatic NADH inhibition of SIRT1 during the daytime plays an important role in energy-sparing in liver under a low-energy state. Whether redox regulation of other sirtuin isoforms (SIRT2–7) could also contribute to tissue-specific promotion of energy conservation during low-energy conditions remains to be explored. Future studies are necessary to elucidate whether other energy-sensing pathways influence the capacity of NADH to regulate the activity of SIRT1 or other sirtuin isoforms during low-energy states in vivo. Our finding that the cytonuclear form of *Lb*NOX drives SIRT1 activity in the nucleus to affect the transcriptional, metabolic and physiological response to TRF-CR suggests that SIRT1 is sensitive to changes in cytosolic NADH and/or to metabolic conditions that cause export of mitochondrial NADH equivalents into the cytosol as occurs during gluconeogenesis^32–35^. Although recent reports suggest that SIRT1 may interact with NADH in the nucleus^68^, continued development of methods that can accurately report subcellular concentrations of reduced/oxidized NAD(H) in vivo will facilitate greater understanding of how redox state across the day under different nutrient conditions may regulate SIRT1 and other sirtuin isoforms^16^.

## Methods

### Animals

All animal procedures were in accordance with guidelines of the Institutional Animal Care and Use Committee at Northwestern University. Mouse protocols approved in this study include IS00003543, IS00007712, IS00001143 and IS00000601. Mice were housed at 23–25 °C in the Center for Comparative Medicine at Northwestern University and maintained under 12:12 h light/dark cycles at 40–60% humidity with ad libitum access to regular chow and water unless otherwise indicated. C57BL/6J mice were purchased from Jackson Laboratories. *Sirt1*^*fx/fx*^ mice harbouring *LoxP* sites surrounding exon 4 of *Sirt1* were from S. Imai (Washington University, St. Louis). *Alb-Cre* mice were purchased from Jackson Laboratories. For liver-specific *Sirt1* knockout studies, *Alb-Cre*; *Sirt1*^*fx/fx*^ mice were used for western blotting and ChIP-Seq, whereas *Sirt1*^*fx/fx*^ mice retro-orbitally injected with AAV8–TBG-iCre were used for transcriptomic, metabolomic and physiological studies. For inducible liver-specific *Bmal1* knockout studies, mice harbouring *LoxP* sites surrounding exon 8 were retro-orbitally injected with AAV8–TBG-iCre. Male mice, 4–6 months old, were used in all experiments, except for studies monitoring fasted body temperature which were performed in 4–6-month-old female mice.

### Caloric restriction studies

Mice were housed individually in cages containing automated, programmable feeder devices (Actimetrics) as described previously^18^. Mice acclimated to feeder cages for 3 days with ad libitum access to food, where the average mouse consumed eleven 300 mg pellets of food each day. During dietary intervention, mice fed regular chow mice received ten pellets of food across the dark period (one 300 mg pellet every 1.2 h), whereas TRF-CR mice received six pellets of food across the dark period (one 300 mg pellet every 2 h). Micronutrient and macronutrient composition of CR food was boosted to compensate for consumption of 40% fewer pellets. Bio-Serv diet F05314, AIN-93M 40% DR was used for CR and Bio-Serv diet F05312 AIN-93M was used for the regular chow. Diet compositions are given in Supplementary Table 1.

### Behavioural analyses

Locomotor activity was analysed in a discrete cohort of TRF-Reg and TRF-CR mice over the duration of the intervention. Mice were housed singly in mouse cages equipped with running wheels in standard 12:12 h light/dark conditions. Twenty-four hour wheel-running activity from each mouse was determined over the duration of the intervention and averaged for each experimental group using ClockLab software (Actimetrics). Activity onset was determined automatically by ClockLab software.

### Ex vivo PER2::LUC measurements

PER2::LUC signal was monitored from SCN of mice as described previously^10^. Briefly, SCN were excised from the hypothalamus of transgenic mice expressing PER2 fused to LUCIFERASE (PER2::LUC) and transferred to a 0.2 µm filter (Millipore) exposed to media (1.2 ml DMEM; Gibco) containing 0.1 mM luciferin sodium salt (Biosynth AG), sodium bicarbonate (352.5 μg ml^−1^), 10 mM HEPES (Gibco), 2 mM l-glutamine, 2% B-27 serum-free supplement (Invitrogen), penicillin (25 U ml^−1^) and streptomycin (20 μg ml^−1^; Gibco). Dishes were sealed, maintained and monitored in a lumicycle (Actimetrics) at 37 °C as described previously^10^.

### Oral glucose tolerance testing

Glucose tolerance tests were performed as described previously^69^. A dose of 1 g kg^−1^ glucose was gavaged per mouse on regular or TRF-CR diets without additional fasting, and blood samples were taken for blood glucose at 0, 5, 15, 60 and 120 min following gavage and for serum insulin at 0, 15 and 30 min following gavage. Blood glucose was measured by hand-held meter and serum insulin was measured by enzyme-linked immunosorbent assay.

### Stable isotope lactate administration

[U-^13^C] lactate at 98% purity (Cambridge Isotope Laboratories) and [U-^13^C] pyruvate at 99% purity (Cambridge Isotope Laboratories) were mixed in a 10:1 ratio to maintain intracellular equilibrium as described previously^70^. Intraperitoneal injections were performed into mice at ZT4 (daytime) to achieve a dose of 1g kg^−1^. Liver was collected after 30 min for MS analysis of hydrophilic metabolites as below. Percentage of each mass isotopomer that was detected for glyceraldehyde-3-phophate/dihydroxyacetone phosphate was quantified.

### NAD^+^ and NADH quantification

NAD^+^ was quantified as described previously^43^. Briefly, cut and weighed tissue was homogenized in perchloric acid (Sigma) in a TissueLyzer (Qiagen). Following neutralization with K_2_CO_3_, supernatant was diluted 1:1 in the mobile phase and analysed by HPLC (Shimadzu) on a Supelco LC_18_ column (Sigma) with an ultraviolet–visible spectroscopy (UV–Vis) detector at 260 nm. NADH was extracted as described previously^20^. Briefly, cut and weighed tissue was gently homogenized by plastic pellet in five volumes of nitrogen-sparged alkaline extraction buffer (25 mM NH_4_Ac, 25 mM NaOH, 50% v/v acetonitrile). Tissue and protein precipitates were pelleted and supernatant was passed through a 0.2 µm HPLC-grade nylon filter. Extract was analysed by HPLC as above with a UV–Vis detector set to 340 nm.

### Mass spectrometry for hydrophilic metabolites

Hydrophilic metabolites were analysed by HPLC–MS/MS as described previously^71^. Briefly, 20–100 mg of weighed tissue was homogenized in 1 ml of cold methanol in the TissueLyzer, centrifuged and the equivalent volume for 10 mg of tissue was dried by SpeedVac, reconstituted in 50% acetonitrile and applied to the HPLC–MS/MS analysis. Data acquisition and analysis were carried out using Xcalibur v.4.1 and Tracefinder v.4.1 software, respectively (ThermoFisher Scientific). Metabolite concentrations within each sample were normalized to total ion count. Data are represented as log_2_(fold change) relative to the control condition as indicated in the text and legends.

### Body temperature analyses

Body temperature was monitored as described previously^10^. Briefly, mice were subcutaneously implanted with wireless temperature probes (IPTT-300) in the dorsal region. After a 1-week recovery, animals were monitored from outside the cage with a hand-held device (DAS-8007-IUS) every 2 h for either 24 or 48 h with minimal disruption to the mice (Bio Medic Data Systems). For analyses of fasted liver-specific *Bmal1* or *Sirt1* knockout female mice, mice were first moved into fresh cages without food at ZT0 (lights on), and body temperature was monitored as above.

### Retro-orbital delivery of AAVs

Retro-orbital delivery of AAVs was performed as described previously^72^. Briefly, *Lb*NOX and control plasmids (Addgene 75285) were cloned into AAV expression vectors under the thyroid-binding globulin (TBG) promoter (to avoid inflammation associated with adenoviral-based methods), packaged into AAVs of serotype 8, purified, and concentrated by Vector Biolabs. AAV8–TBG–iCre and AAV8–TBG–green fluorescent protein (GFP) plasmids were purchased from Vector Biolabs (catalogue numbers: VB1724 and VB1743, respectively). Following isofluorane anaesthesia, mice were injected retro-orbitally with 1 × 10^11^ genome copies (GC) of the respective virus in 100 µl. For mice expressing multiple AAVs, mice were co-injected with a 100 µl mixture of both viruses to achieve 5 × 10^10^ GC of iCre/GFP AAVs and 1 × 10^11^ GC of *Lb*NOX/null AAVs. Mice were monitored acutely for recovery to anaesthesia then given 3 days to recover from AAV8 administration. Mice were then transferred to experimental chambers and allowed to acclimate to that environment for 2 weeks before the start of dietary interventions.

### RNA sequencing and analysis

RNA was isolated from 10–20 mg of liver tissue using the Zymo Direct-Zol RNA miniprep kit as described described^10^. RNAs with an RNA integrity number > 8 were used for library preparation, which was constructed from 250 ng of RNA with the NEBNext RNA Ultra Directional library prep kit (NEB). Seventy-five base pair single-end sequencing was performed on a NextSeq 500 sequencer with a high output Illumina flow cell (FC-404-2002) and analysed as described previously^10^. Briefly, sequences were aligned to the mm10 genome with STAR (v.2.5.2), assigned to Ensembl features (GRCm38.vM12) with subread:featureCounts (v.1.5.1), and analysed for differential expression at each time point in DESeq2 (v.1.24.0). The FDR-adjusted *P*-value cut-off for significance was set to 0.05. Gene ontology Kyoto Encyclopedia of Genes and Genomes (KEGG) analyses were performed with the HOMER findGO command, and principal components analyses were performed by DESeq2 on variance stabilization transformed RNA-Seq data for genes differentially expressed by diet. Known motif analysis was performed by HOMER (v.4.8.3) using the JASPAR database of DNA-binding motifs for regions of open chromatin previously identified by ATAC sequencing in liver of ad libitum mice at ZT4 that annotate to genes listed as in results and legends^10^. For comparison of genomics datasets, quadrant plots were utilized in which log_2_(fold change) in gene expression from TRF-CR (relative to TRF-Reg) was compared with the log_2_(fold change) in gene expression from the given dataset using genes that are significantly differentially expressed by diet. Genes within each quadrant were counted and expressed as a fraction of the total. For comparison with outside datasets involving *Bmal1*^−/−^ and *Ppara*^−/−^, a fold change cut-off of ±0.5 was applied. *Lb*NOX and iCre expression were determined by using Bowtie2 (v.2.2.4) to align to their respective sequences and dividing by ten million reads of each library. *Sirt1* knockout was confirmed by taking the ratio of reads within the exon flanked by *LoxP* sites (exon 4) and exon 9 of *Sirt1* using subread:featureCounts set to count by exon.

### ChIP sequencing and analysis

Liver was processed for ChIP-Seq as described previously^10^. Briefly, liver was double cross-linked, nuclei isolated with hypotonic buffer and needle lysis, and chromatin sheared by sonication (Diagenode). Chromatin was diluted 1:10 in dilution buffer (0.01% SDS, 1.1% Triton X-100, 167 mM NaCl, 1.2 mM EDTA pH 8, 1.67 mM Tris–HCl pH 8), and immunoprecipitations were performed with 15 µg of anti-BMAL1 (Millipore, catalogue number: ABE2599) or FOXO1 (Abcam, catalogue number: ab39670) antibodies, and secondary-conjugated, bovine serum albumin-blocked paramagnetic beads pulled-down protein/DNA complexes. Chromatin was de-cross-linked and purified before library prep (NEBNext Ultra II library prep kit). 2 ng of each replicate was processed into libraries, which were size selected to 200–600 bp before polymerase chain reaction amplification and pooled for sequencing as in ref. ^10^. Bowtie2 (v.2.2.4) was used to align sequencing data to the mm10 genome with standard parameters. Peaks were called using the HOMER (v.4.8.3) findPeaks command with the settings -style factor, -size 275, -fragLength 250. For scatter plots, the tag-density for individual peaks for each replicate as described was quantified by HOMER annotatePeaks with setting -size given, and averaged. For quadrant plots, a fold change cut-off of ±0.5 was applied and the number of peaks within each quadrant was counted.

### Solid-phase peptide synthesis

Peptide substrates were synthesized on Fmoc-rink amide 4-methylbenzhydrylamine resin (AnaSpec. Inc.) as described previously^73^. Briefly, the N-terminal fluorenylmethyloxycarbonyl was deprotected with 20% piperidine in *N*,*N*-dimethylformamide, filtered and washed with *N*,*N*-dimethylformamide before coupling with PyBop and *N*-methylmorpholine. Following filtration and washing of the coupled resin, the cleavage cocktail (95% trifluoroacetic acid, 2.5% H_2_O and 2.5% triethylsilane) was applied and solutions were evaporated under nitrogen, dissolved in water and lyophilized before purification by HPLC.

### Protein expression

SIRT1 (amino acids 193–747) was produced as reported previously^74^ and expressed in BL21 DE3 using a pTriEx-based expression vector. Cells were grown in 2XTY broth supplied with 100 µg ml^−1^ carbenicillin at 37 °C until the optical density reached ~0.6, then induced with isopropyl-β-d-thiogalactoside (1 mM) and ZnSO_4_ (0.2 mM). Cells were harvested after 3 h of incubation by centrifugation and then lysed by lysozyme in lysis buffer (50 mM Tris pH 8, 5 mM *β*-mercaptoethanol and 5% glycerol). His-tagged SIRT1 was then purified using standard immobilized metal affinity chromatography. The eluted protein was then dialysed using a Slide-A-Lyzer dialysis cassette 20K MWCO (Millipore) in dialysis buffer (50 mM Tris pH 8, 150 mM KCl, 5 mM *β*-mercaptoethanol and 5% glycerol) and then concentrated using a 30 kDa centrifugal filter (Millipore). Purified samples were analysed by SDS–PAGE.

### Sirtuin reactions

Recombinant SIRT1, NAD^+^ and NADH were first diluted in KDAC buffer (25 mM Tris pH 8.0, 137 mM NaCl, 2.7 mM KCl, 1 mM MgCl_2_ in deionized ultrafiltered water). Some 2 µl of SIRT solution with 1 μl of NAD^+^ and 1 μl NADH were applied to each reaction well, to give final enzyme concentrations of 30–100 nM, with NAD^+^ and NADH as indicated. Then 1 μl of the acetylated peptide substrate was added at a final concentration of the peptide’s Michaelis constant (*K*_M_) to initiate the reaction. The reaction plate was incubated at 37 °C for 15–20 min (within the linear response range), then quenched with excess of the deacetylase inhibitor nicotinamide (50 mM). *K*_i(NADH)_ and K_M(NAD+)_ were derived by fitting the initial velocity data into a nonlinear regression competitive inhibitor model in Graphpad Prism software (v.9.2.0). We derived the kinetic constants assuming the kinetics of the thiol form of substrate and the disulfide form are the same. Lineweaver–Burk transformation was determined by inverting data and conducting linear curve-fitting.

### SAMDI-MS

Small volumes of each reaction (2 μl) were transferred onto an array plate having 384 gold islands modified with a maleimide-presenting monolayer to allow immobilization of the peptide substrate and product. The monolayers were then rinsed with deionized ultrafiltered water and ethanol, dried under nitrogen and treated with matrix (2,4,6-trihydroxyacetophenone, 20 mg ml^−1^ in acetone). The monolayers were analysed with a 4800 MALDI-TOF/TOF mass spectrometer (Applied Biosystems) to obtain a mass spectrum for each spot as described previously^73^.

### Cell culture

Mouse embryonic fibroblasts or HEK-293T cells (Takara 632180) were cultured in high-glucose DMEM (Corning) containing 1 mM pyruvate and supplemented with 10% foetal bovine serum (Denville) and penicillin/streptomycin (Corning). Then 50 mM of lactate or pyruvate (Sigma-Aldrich) was supplemented into the cell culture media in the presence or absence of 1 µM trichostatin A for 3 h before collection. Lipofectamine P3000 (Invitrogen) was used for transfection experiments.

### Western blotting

Protein was extracted with RIPA buffer from mouse embryonic fibroblasts or liver in the presence of protease inhibitors, trichostatin A, nicotinamide (Sigma-Aldrich) and phosphatase inhibitors (Roche), as described previously^43^. Protein extracts were subjected to SDS–PAGE, transferred to nitrocellulose membranes and probed with primary antibodies specific for acetyl-lysine targets regulated by SIRT1 (H3K9-Ac (Abcam, catalogue number: ab8898); H4K16-Ac (Millipore, catalogue number: 07-329); FOXO1(K242, K245, K262)-Ac (Santa Cruz, catalogue number: sc374427); p53(K379)-Ac (Cell Signaling, catalogue number: 2570)), as well as total levels of H3 (Cell Signaling, catalogue number: 9715), H4 (Cell Signaling, catalogue number: 13919), FOXO1 (Santa Cruz, catalogue number: sc11350), p53 (Cell Signaling, catalogue number: 2524), SIRT1 (Millipore, catalogue number: 07-131), FLAG (Sigma, catalogue number: M8823) and ACTIN (Cell Signaling, catalogue number: 4970) at a dilution of 1:1,000. Signal was quantified relative to total H3 or ACTIN by performing background-corrected densitometry in ImageJ (v.2.0.0). Uncropped western blot scans labelled with molecular weight markers are presented in the Source Data files.

### Acyl-carnitine quantitation

Acyl-carnitine measurements were made by flow-injection MS/MS using sample preparation methods described previously^27,28^. Data was acquired using a Waters TQD mass spectrometer equipped with an AcquityTM UPLC system and controlled by MassLynx 4.1 operating system (Waters).

### Immunohistochemistry

Immunohistochemistry was performed with FLAG antibody (Sigma). Briefly, livers were fresh-fixed in 4% paraformaldehyde before embedding in OCT compound (Tissue Tek) and freezing at −80 °C. Liver slices (20 µm thick) were prepared on a cryostat and stained with FLAG antibody at 1:100 dilution before bright-field colour imaging at ×4 magnification.

### Public genomics datasets used in this study

RNA-Seq data from liver of ad libitum PPARα controls: GSE118787. RNA-seq data from liver of WT and *Bmal1*^−*/*−^ mice: GSE133989.

### Quantitation and statistical analysis

Throughout the article ‘log_2_(fold change (TRF-CR))’ indicates TRF-CR relative to TRF-Reg for null mice unless a different genotype is indicated; ‘log_2_(fold change (*Lb*NOX in TRF-CR))’ indicates TRF-CR, *Lb*NOX relative to TRF-CR, null; ‘log_2_(fold change (*Lb*NOX in TRF-CR, *L-Sirt1*^−*/*−^))’ indicates TRF-CR, *L-Sirt1*^−*/*−^, *Lb*NOX relative to TRF-CR, *L-Sirt1*^−*/*−^, null; ‘log_2_(fold change (*Bmal1*^−*/*−^))’ indicates *Bmal1*^−*/*−^ relative to wild-type littermates; and ‘log_2_(fold change (*Ppara*^−*/*−^))’ indicates *Ppara*^−*/*−^ relative to wild-type littermates. Statistical analysis was performed with unpaired two-tailed Student’s *t* test in Microsoft Excel (v.16.16.16) unless noted otherwise. Data are represented as mean ± s.e.m. unless noted otherwise. Biologically independent replicates in each experiment are noted in the figure legends as the ‘*n*’ specific to each group. Only one ‘*n*’ is provided if the number of independent biological replicates is the same across groups. Quadrant plots were analysed by binomial test. Box and whisker plots depict the following: line, median; box limits, first and third quartiles; whiskers, 10th and 90th percentiles; outliers, not shown. Differences were considered significant when *P* < 0.05 unless noted otherwise. Body temperature rhythms were analysed by two-way analysis of variance, and average body temperature was analysed by two-way Student’s *t* test using an average of all data points shown. Differential amino acids were determined by performing multiple unpaired, two-tailed *t* tests between TRF-CR and *Lb*NOX in TRF-CR using the Benjamini and Hochberg FDR approach set to 5%. The effect of TRF-CR on glucose tolerance and distribution of mass isotopomers of glyceraldehyde-3-phophate/dihydroxyacetone phosphate was analysed by two-way analysis of variance. Differentially expressed liver TRF-CR genes, liver metabolites and serum acyl-carnitines that require SIRT1 for the effect of *Lb*NOX were those that demonstrated either: (1) log_2_(fold change (TRF-CR)) > 0 and log_2_(fold change (*Lb*NOX in TRF-CR)) < 0 and log_2_(fold change (*Lb*NOX in TRF-CR, *Sirt1*^−*/*−^)) >0.5 × log_2_(fold change (*Lb*NOX in TRF-CR)); or (2) log_2_(fold change (TRF-CR)) < 0 and log_2_(fold change (*Lb*NOX in TRF-CR)) > 0 and log_2_(fold change (*Lb*NOX in TRF-CR, *Sirt1*^−*/*−^)) < 0.5 × log_2_(fold change (*Lb*NOX in TRF-CR)).

### Reporting Summary

Further information on research design is available in the Nature Research Reporting Summary linked to this article.

## Supplementary information

Reporting Summary

Supplementary Table 1 Nutrient composition of regular and CR diets.

Supplementary Table 2 LC–MS of amino acids in TRF-CR and *Lb*NOX livers.

Supplementary Table 3 Genes, metabolites and acyl-carnitines requiring SIRT1 for effect of NADH oxidation during TRF-CR.

## Data Availability

Data generated in this study are publicly available in the GEO repository (GSE151281). We also utilized publicly accessible RNA-seq data from GEO repositories GSE133989 and GSE118787. JASPAR databases are found at http://jaspar.genereg.net/search?q=&collection=CORE&tax_group=vertebrates. Correspondence and requests for materials should be addressed to Joseph Bass (j-bass@northwestern.edu). Source data are provided with this paper.

## Source data

Source Data Fig. 3 Unprocessed western blots from Fig. 3c–e.
Source Data Extended Data Fig. 3 Unprocessed western blots from Extended Data Fig. 3g.
