# Supplementary material for: NADH inhibition of SIRT1 links energy state to transcription during time-restricted feeding
Source: Nat Metab. 2021 Dec 13;3(12):1621–32. doi: 10.1038/s42255-021-00498-1 (PMC8688143; doi:10.1038/s42255-021-00498-1)
Supplement: Supplementary file 1 — Reporting Summary [file 42255_2021_498_MOESM1_ESM.pdf]

nature research

Corresponding author(s): Joseph Bass

Last updated by author(s): 10/6/2021

Reporting Summary

Nature Research wishes to improve the reproducibility of the work that we publish. This form provides structure for consistency and transparency in reporting. For further information on Nature Research policies, see our Editorial Policies and the Editorial Policy Checklist.

Statistics

For all statistical analyses, confirm that the following items are present in the figure legend, table legend, main text, or Methods section.

n/a	Confirmed	
☐	☒	The exact sample size (n) for each experimental group/condition, given as a discrete number and unit of measurement
☐	☒	A statement on whether measurements were taken from distinct samples or whether the same sample was measured repeatedly
☐	☒	The statistical test(s) used AND whether they are one- or two-sided<br>*Only common tests should be described solely by name; describe more complex techniques in the Methods section.*
☐	☒	A description of all covariates tested
☐	☒	A description of any assumptions or corrections, such as tests of normality and adjustment for multiple comparisons
☐	☒	A full description of the statistical parameters including central tendency (e.g. means) or other basic estimates (e.g. regression coefficient) AND variation (e.g. standard deviation) or associated estimates of uncertainty (e.g. confidence intervals)
☐	☒	For null hypothesis testing, the test statistic (e.g. F, t, r) with confidence intervals, effect sizes, degrees of freedom and P value noted<br>*Give P values as exact values whenever suitable.*
☒	☐	For Bayesian analysis, information on the choice of priors and Markov chain Monte Carlo settings
☐	☒	For hierarchical and complex designs, identification of the appropriate level for tests and full reporting of outcomes
☒	☐	Estimates of effect sizes (e.g. Cohen's d, Pearson's r), indicating how they were calculated

Our web collection on statistics for biologists contains articles on many of the points above.

Software and code

Policy information about availability of computer code

Data collection	*Provide a description of all commercial, open source and custom code used to collect the data in this study, specifying the version used OR state that no software was used.*
Data analysis	We used publicly-accessible software and standard parameters to analyze our data. For RNA-Seq, STAR (v2.5.2), subread:featureCounts (v1.5.1), DESeq2 (v1.24.0), and HOMER (v4.8.3) were used. For ChIP-Seq, Bowtie2 (v2.2.4) and HOMER (v4.8.3) were used. ClockLab software (Version 6, Actimetrics) was used to analyze wheel running activity, and Tracefinder (v4.1, Thermo Fisher Scientific) was used to analyze metabolite concentrations following HPLC-MS/MS. All other analyses were performed in Microsoft Excel (v16.16.16) and Graphpad PRISM (v9.2.0). Western blot images were quantified with ImageJ (v2.0.0).

For manuscripts utilizing custom algorithms or software that are central to the research but not yet described in published literature, software must be made available to editors and reviewers. We strongly encourage code deposition in a community repository (e.g. GitHub). See the Nature Research guidelines for submitting code & software for further information.

Data

Policy information about availability of data

All manuscripts must include a data availability statement. This statement should provide the following information, where applicable:

- Accession codes, unique identifiers, or web links for publicly available datasets
- A list of figures that have associated raw data
- A description of any restrictions on data availability

Data and Materials Availability: Data generated in this study are publicly available in the GEO repository (GSE151281). We also utilized publicly-accessible RNA-seq data from GEO repositories GSE133989 and GSE118787. JASPAR databases are found at http://jaspar.genereg.net/search?

q=&collection=CORE&tax_group=vertebrates . Source data are provided with this paper. Correspondence and requests for materials should be addressed to Joseph Bass (j-bass@northwestern.edu). Reprints and permissions information is available at www.nature.com/reprints.

Field-specific reporting

Please select the one below that is the best fit for your research. If you are not sure, read the appropriate sections before making your selection.

☒ Life sciences ☐ Behavioural & social sciences ☐ Ecological, evolutionary & environmental sciences

For a reference copy of the document with all sections, see nature.com/documents/nr-reporting-summary-flat.pdf

Life sciences study design

All studies must disclose on these points even when the disclosure is negative.

Sample size	Power calculations were performed to identify the minimum number of mice for each assay that would achieve 90% power given measured differences in means and standard deviations for each assay in previous work in our lab as in (Levine DC et al, Molecular Cell, 2020).
Data exclusions	We used pre-determined criteria to exclude outliers including IQR*1.5 rule or PCA clustering at a substantial distance away from other replicates. One replicate from the AAV8-LbNOX-transduced, TRF-CR group was excluded as a result of being identified as an outlier by the above criteria in assays measuring protein and RNA levels of LbNOX, NADH, and also in clustering of RNA-seq data.
Replication	All findings in mice were successfully replicated at each attempt and were replicated in at least one independent cohort containing multiple mice. In cell-based studies, results were repeated in at least 3 independent experiments collected and assayed on different days.
Randomization	Littermates were distributed evenly into each group.
Blinding	Code-names were applied to samples to blind researchers during sample processing at the bench and during data analysis.

Reporting for specific materials, systems and methods

We require information from authors about some types of materials, experimental systems and methods used in many studies. Here, indicate whether each material, system or method listed is relevant to your study. If you are not sure if a list item applies to your research, read the appropriate section before selecting a response.

Materials & experimental systems

n/a	Involved in the study	
☐	☒	Antibodies
☐	☒	Eukaryotic cell lines
☒	☐	Palaeontology and archaeology
☐	☒	Animals and other organisms
☒	☐	Human research participants
☒	☐	Clinical data
☒	☐	Dual use research of concern

Methods

n/a	Involved in the study	
☐	☒	ChIP-seq
☒	☐	Flow cytometry
☒	☐	MRI-based neuroimaging

Antibodies

Antibodies used	All antibodies were used at a dilution of 1:1000. H3K9-Ac (Abcam ab8898), H4K16-Ac (Millipore 07-329), FOXO1(K242,K245,K262)-Ac (Santa Cruz sc-49437), p53(K379)-Ac (Cell Signaling 2570), H3 (Cell Signaling 9715), H4 (Cell Signaling 13919), FOXO1 (Santa Cruz sc374427), p53 (Cell Signaling 2524), SIRT1 (Millipore 07-131), FLAG (Sigma M8823), ACTIN (Cell Signaling 4970), FOXO1 ChIP (Abcam ab39670), and BMAL1 ChIP (Millipore ABE2599).
Validation	Antibodies chosen have been widely published, contain validation statements in the vendor websites, and/or were validated by the Antibody Validation Database (Park Lab). For the FOXO1 Abcam ChIP antibody, ChIP-seq datasets were validated by confirming expected phenotypes (i.e. increased binding to known targets following prolonged fasting and in insulin receptor knockouts), by performing unbiased DNA motif analysis with HOMER, and by comparing results to a Santa Cruz FOXO1 antibody (PMID: 30532187) using peaks from our antibody as reference.

Eukaryotic cell lines

Policy information about cell lines

Cell line source(s)	Immortal wild type mouse embryonic fibroblasts were generated in lab as previously described (Levine DC et al, Molecular Cell, 2020). HEK293T cells were obtained from Takara (632180).

Authentication	None of the cell lines were authenticated.
Mycoplasma contamination	MEF and HEK293 cells used in this study have tested negative for mycoplasma contamination.
Commonly misidentified lines (See ICLAC register)	Our cell lines are not commonly misidentified.

Animals and other organisms

Policy information about studies involving animals; ARRIVE guidelines recommended for reporting animal research

Laboratory animals	4-6 mo old male C57B6/J mice were used in TRF-Reg and TRF-CR mice +/- LbNox. We also utilized liver-specific Sirt1 KO mice (generated either by crossing Sirt1 fx/fx mice with Alb-Cre mice or by injecting Sirt1 fx/fx mice with AAV8-TBG-iCre) and liver-specific Bmal1 KO mice (generated by injecting Bmal1 fx/fx mice with AAV8-TBG-iCre). Male 4-6 month old male mice were used in all experiments, except for studies monitoring fasted body temperature which were performed in 4-6 month old female mice.
Wild animals	No wild animals were used in this study.
Field-collected samples	No samples were collected in the field in this study.
Ethics oversight	All animal procedures were in accordance with guidelines of the Institutional Animal Care and Use Committee at Northwestern University. Mouse protocols approved in this study include: IS00003543, IS00007712, IS00001143, and IS00000601.

Note that full information on the approval of the study protocol must also be provided in the manuscript.

ChIP-seq

Data deposition

☒ Confirm that both raw and final processed data have been deposited in a public database such as GEO.

☒ Confirm that you have deposited or provided access to graph files (e.g. BED files) for the called peaks.

Data access links<br>*May remain private before publication.*	Data in this study is publicly available in the GEO repository (GSE151281). Correspondence and requests for materials should be addressed to Joseph Bass (j-bass@northwestern.edu).
Files in database submission	LbCR4-RNA.NULL.Reg.1.fastq.gz<br>LbCR4-RNA.NULL.Reg.2.fastq.gz<br>LbCR4-RNA.NULL.Reg.3.fastq.gz<br>LbCR4-RNA.NULL.Reg.4.fastq.gz<br>LbCR4-RNA.NULL.Reg.5.fastq.gz<br>LbCR4-RNA.LbNOX.Reg.1.fastq.gz<br>LbCR4-RNA.LbNOX.Reg.2.fastq.gz<br>LbCR4-RNA.LbNOX.Reg.3.fastq.gz<br>LbCR4-RNA.LbNOX.Reg.4.fastq.gz<br>LbCR4-RNA.LbNOX.Reg.5.fastq.gz<br>LbCR4-RNA.LbNOX.Reg.6.fastq.gz<br>LbCR4-RNA.NULL.CR.1.fastq.gz<br>LbCR4-RNA.NULL.CR.2.fastq.gz<br>LbCR4-RNA.NULL.CR.3.fastq.gz<br>LbCR4-RNA.NULL.CR.4.fastq.gz<br>LbCR4-RNA.NULL.CR.5.fastq.gz<br>LbCR4-RNA.NULL.CR.6.fastq.gz<br>LbCR4-RNA.LbNOX.CR.1.fastq.gz<br>LbCR4-RNA.LbNOX.CR.2.fastq.gz<br>LbCR4-RNA.LbNOX.CR.3.fastq.gz<br>LbCR4-RNA.LbNOX.CR.4.fastq.gz<br>LbCR4-RNA.LbNOX.CR.5.fastq.gz<br>LbCR4-FOXO1.NULL.Reg.1.fastq.gz<br>LbCR4-FOXO1.NULL.Reg.2.fastq.gz<br>LbCR4-FOXO1.LbNOX.Reg.1.fastq.gz<br>LbCR4-FOXO1.LbNOX.Reg.2.fastq.gz<br>LbCR4-FOXO1.LbNOX.Reg.3.fastq.gz<br>LbCR4-FOXO1.NULL.CR.1.fastq.gz<br>LbCR4-FOXO1.NULL.CR.2.fastq.gz<br>LbCR4-FOXO1.NULL.CR.3.fastq.gz<br>LbCR4-FOXO1.LbNOX.CR.1.fastq.gz<br>LbCR4-FOXO1.LbNOX.CR.2.fastq.gz<br>S1CR4-FOXO1.CTRL.Reg.1.fastq.gz<br>S1CR4-FOXO1.CTRL.Reg.2.fastq.gz<br>S1CR4-FOXO1.CTRL.CR.1.fastq.gz<br>S1CR4-FOXO1.CTRL.CR.2.fastq.gz<br>S1CR4-FOXO1.LSKO.Reg.1.fastq.gz<br>S1CR4-FOXO1.LSKO.Reg.2.fastq.gz

S1CR4-FOXO1.LSKO.CR.1.fastq.gz
S1CR4-FOXO1.LSKO.CR.2.fastq.gz
S1CR4-INPUT.CTRL.Reg.1.fastq.gz
S1CR4-INPUT.CTRL.CR.1.fastq.gz
S1CR4-INPUT.LSKO.Reg.1.fastq.gz
S1CR4-INPUT.LSKO.CR.1.fastq.gz
LbCR4-INPUT-NULL.Reg.1.fastq.gz
LbCR4-INPUT-LbNOX.Reg.1.fastq.gz
LbCR4-INPUT-NULL.CR.1.fastq.gz
LbCR4-INPUT-LbNOX.CR.1.fastq.gz
S1CR4-BMAL1-CTRL.Reg.1.fastq.gz
S1CR4-BMAL1-CTRL.Reg.2.fastq.gz
S1CR4-BMAL1-CTRL.Reg.3.fastq.gz
S1CR4-BMAL1-LSKO.Reg.1.fastq.gz
S1CR4-BMAL1-LSKO.Reg.2.fastq.gz
S1CR4-BMAL1-LSKO.Reg.3.fastq.gz
S1CR4-BMAL1-CTRL.CR.1.fastq.gz
S1CR4-BMAL1-CTRL.CR.2.fastq.gz
S1CR4-BMAL1-CTRL.CR.3.fastq.gz
S1CR4-BMAL1-LSKO.CR.1.fastq.gz
S1CR4-BMAL1-LSKO.CR.2.fastq.gz
S1CR4-BMAL1-LSKO.CR.3.fastq.gz
LbCR4-BMAL1-NULL.Reg.1.fastq.gz
LbCR4-BMAL1-NULL.Reg.2.fastq.gz
LbCR4-BMAL1-NULL.Reg.3.fastq.gz
LbCR4-BMAL1-LbNOX.Reg.1.fastq.gz
LbCR4-BMAL1-LbNOX.Reg.2.fastq.gz
LbCR4-BMAL1-LbNOX.Reg.3.fastq.gz
LbCR4-BMAL1-NULL.CR.1.fastq.gz
LbCR4-BMAL1-NULL.CR.2.fastq.gz
LbCR4-BMAL1-NULL.CR.3.fastq.gz
LbCR4-BMAL1-LbNOX.CR.1.fastq.gz
LbCR4-BMAL1-LbNOX.CR.2.fastq.gz
PPARa-RNA.KO.AdLib.1.fastq.gz
PPARa-RNA.KO.AdLib.2.fastq.gz
PPARa-RNA.KO.AdLib.3.fastq.gz
PPARa-RNA.KO.AdLib.4.fastq.gz
LbCRKO4-RNA.NULL.CR.1.fastq.gz
LbCRKO4-RNA.NULL.CR.2.fastq.gz
LbCRKO4-RNA.NULL.CR.3.fastq.gz
LbCRKO4-RNA.NULL.CR.4.fastq.gz
LbCRKO4-RNA.NULL.CR.5.fastq.gz
LbCRKO4-RNA.NULL.CR.6.fastq.gz
LbCRKO4-RNA.LbNOX.CR.1.fastq.gz
LbCRKO4-RNA.LbNOX.CR.2.fastq.gz
LbCRKO4-RNA.LbNOX.CR.3.fastq.gz
LbCRKO4-RNA.LbNOX.CR.4.fastq.gz
LbCRKO4-RNA.LbNOX.CR.5.fastq.gz
LbCRKO4-RNA.LbNOX.CR.6.fastq.gz

Genome browser session
(e.g. UCSC)

no longer applicable

Methodology

Replicates

Exact replicate numbers are stated in the figure legends. Typically, separate ChIPs, library preps, and analyses were performed for each of 2-3 mice with signal averaged during plotting. In some cases the number of mice was higher. Replicates were tested for agreement by assessing deviation from the diagonal in a scatterplot.

Sequencing depth

For ChIP-Seq, we performed 75 base pair, single-end sequencing to generate ~15-25M reads per sample, of which, ~70-80% aligned to the mm10 genome exactly 1 time.

Antibodies

Immunoprecipitations were performed in 1/4 liver with 15 µg of anti-BMAL1 (Millipore ABE2599) or FOXO1 (Abcam ab39670).

Peak calling parameters

For each ChIP, peaks were called using the HOMER findPeaks command with settings -style factor, -size 275, -fragLength 250. We used the HOMER defaults enrichment parameters which include a 4-fold enrichment over background (input), a 4-fold enrichment over local tags, and an FDR cutoff of 1%. Pooled inputs for each condition were used as background. Peaks over input were identified in each replicate, then merged. With these parameters, we identified ~550 FOXO1 peaks, similar to what has been reported previously (PMID: 30532187) and ~35,000 BMAL1 peaks, similar to what has been reported previously (PMID: 32369735).

Data quality

High quality data was confirmed by visually analyzing bigwig files in UCSC, performing motif-finding analysis with HOMER, confirming previously published phenotypes and binding locations, using Pippin Prep library size selection, Agilent Bioanalyzer, and standard Illumina QC parameters. We used the HOMER defaults that include a 4-fold enrichment over background (input), a 4-fold enrichment over local tags, and an FDR cutoff of 1%. With these parameters, we identified ~550 FOXO1 peaks, similar to what has been reported previously (PMID: 30532187), and ~35,000 BMAL1 peaks, similar to what has been reported previously (PMID: 32369735).

Software

Bowtie2 (v2.2.4) was used to align sequencing data to the mm10 genome with standard parameters. For each ChIP, peaks were called using the HOMER findPeaks command with settings -style factor, -size 275, -fragLength 250. For scatter plots, tag-density for individual peaks for each replicate as described was quantified by HOMER annotatePeaks with setting -size given, and averaged. Known motif analysis was performed by HOMER using the JASPAR database of DNA binding motifs for peaks identified in results and legends. Gene ontology analysis was similarly performed with HOMER.
