# Supplementary figures and images for: NADH inhibition of SIRT1 links energy state to transcription during time-restricted feeding
Source: Nat Metab. 2021 Dec 13;3(12):1621–32. doi: 10.1038/s42255-021-00498-1 (PMC8688143; doi:10.1038/s42255-021-00498-1)

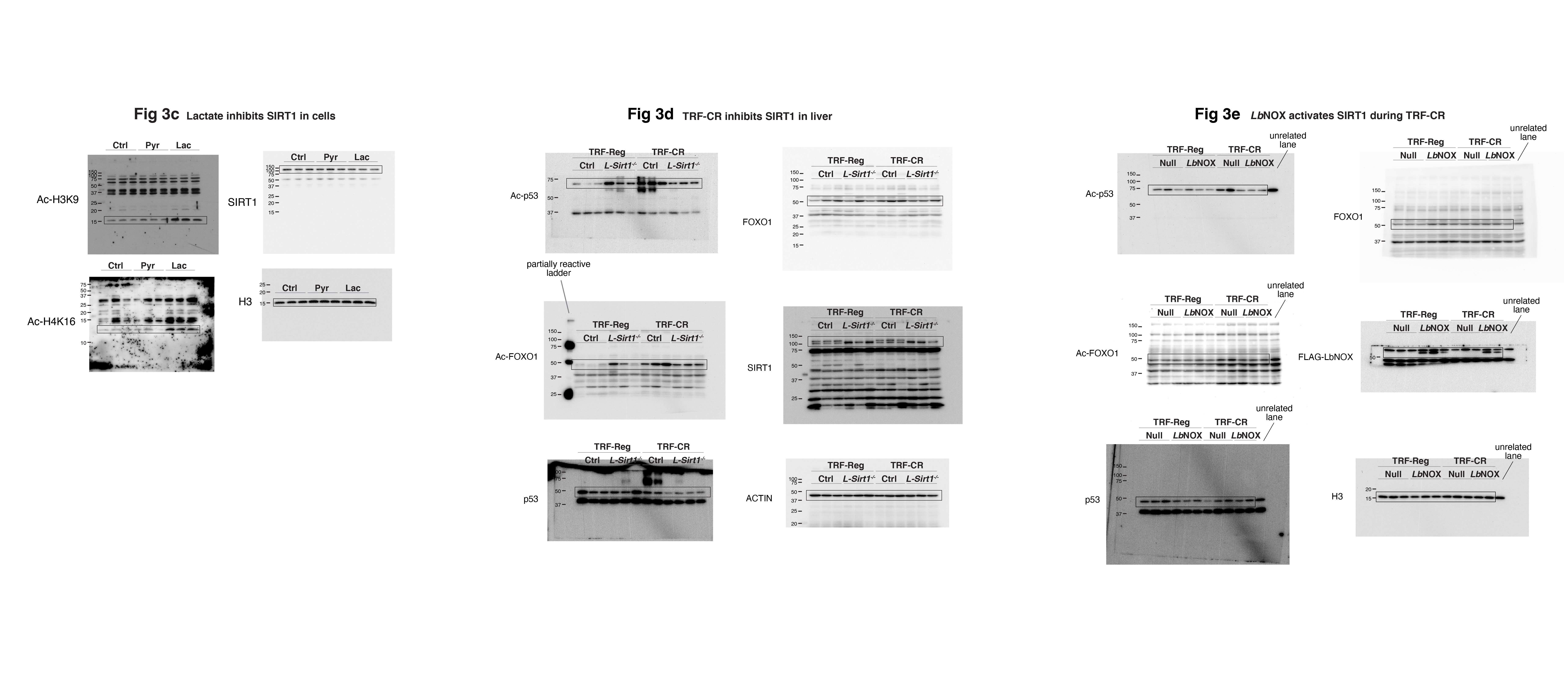

Supplement: Source Data Fig. 3 — Unprocessed western blots from Fig. 3c–e. [file 42255_2021_498_MOESM5_ESM.jpg]

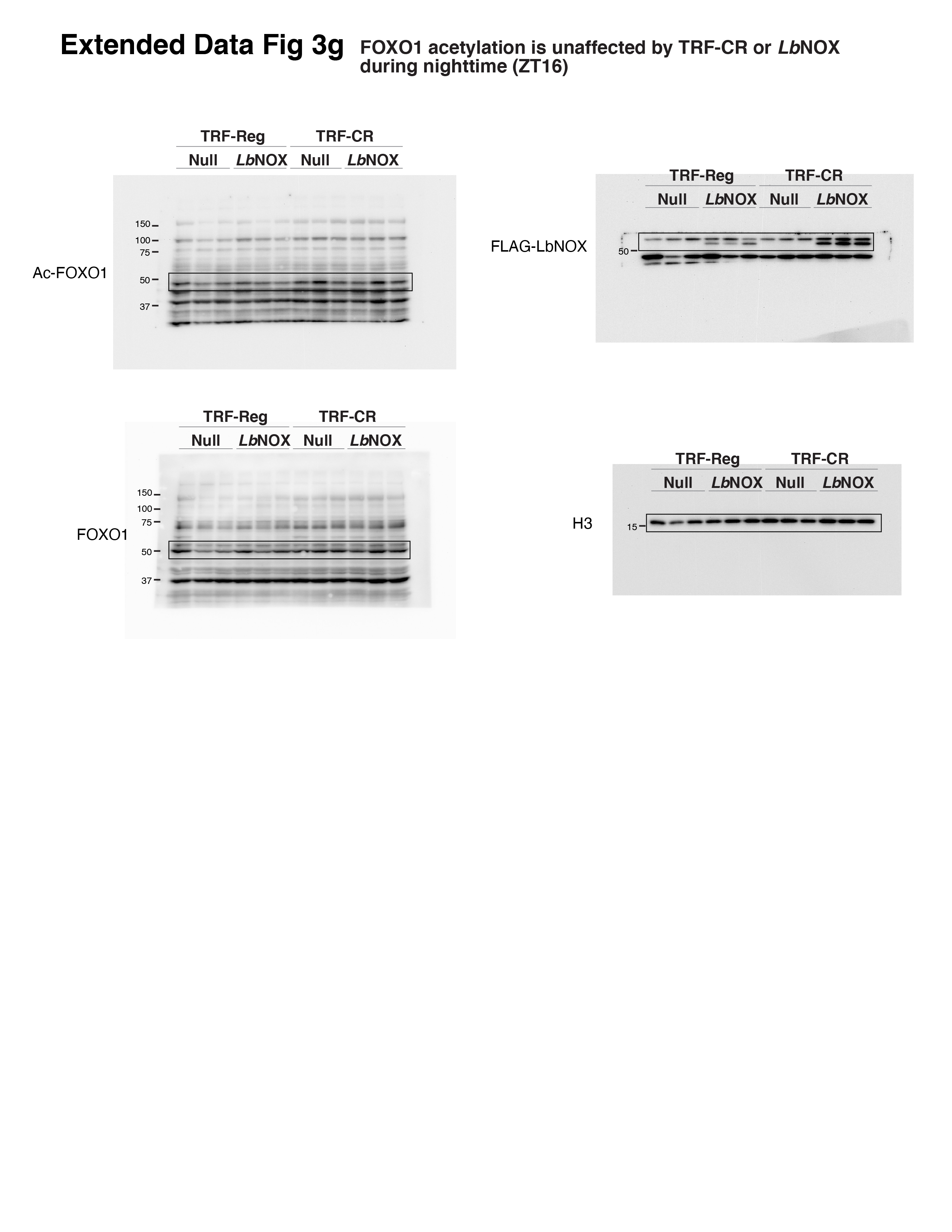

Supplement: Source Data Extended Data Fig. 3 — Unprocessed western blots from Extended Data Fig. 3g. [file 42255_2021_498_MOESM6_ESM.jpg]
